# Design and development of a modular wrist rehabilitation robot with impedance control and gravity compensation

**DOI:** 10.1038/s41598-025-31185-w

**Published:** 2025-12-06

**Authors:** Zahra Baradaran Ghaffari, Majid Sadedel, Majid Moghaddam, Hussain Orooji Khouzani

**Affiliations:** Department of Mechanical Engineering, Tarbiat Modares University, Tehran, Iran

**Keywords:** Rehabilitation robotic, Wrist and forearm rehabilitation, Modular robots, Impedance controller, Weight compensator, Engineering, Mathematics and computing

## Abstract

The number of stroke patients is steadily increasing, highlighting the critical need for rehabilitation to restore motor function. The shortage of rehabilitation specialists and the repetitive nature of traditional exercises underscore the advantages of robotic systems. However, many existing rehabilitation robots are not adaptable to various therapeutic requirements. Modular robots, with their reconfigurability and ease of assembly, provide a practical solution. This study presents the design and development of a modular wrist rehabilitation robot, consisting of three identical modules that provide the necessary degrees of freedom for wrist movement. The modular structure allows for easy assembly, adjustment for different hand sizes, and adaptability to various configurations. To improve control performance, an impedance control strategy was implemented along with a gravity compensation method. Simulation results show that impedance control alone resulted in an RMS error of approximately 7 degrees, while adding gravity compensation reduced the maximum error to 0.79 degrees. The proposed controller was implemented on the robot and validated through experimental tests with individuals. The results confirmed that impedance control effectively facilitates interaction between the robot and the user, demonstrating the system’s potential for improving rehabilitation outcomes.

**Supplementary Information:**

The online version contains supplementary material available at 10.1038/s41598-025-31185-w.

## Introduction

 Stroke, a leading cause of long-term disability, can significantly impair motor functions, particularly in the upper limbs, necessitating effective rehabilitation methods. Traditional rehabilitation exercises often require repetitive movements under the supervision of therapists, which can be time-consuming and physically demanding for both patients and caregivers. To address these challenges, robotic rehabilitation has emerged as a promising solution, offering precision, consistency, and the ability to provide intensive training without fatigue^1^. Among various robotic rehabilitation devices, wrist rehabilitation robots play a crucial role due to the essential function of the wrist in performing daily activities such as grasping, lifting, and manipulating objects. The wrist’s complex structure requires a rehabilitation device capable of providing multiple degrees of freedom to accommodate various movements.

Robotic rehabilitation has significantly advanced the field of physical therapy, particularly concerning wrist rehabilitation. Early developments, such as the MIT-MANUS robot, laid the foundation for robotic rehabilitation by introducing adaptive impedance control tailored to patients’ specific needs^2^. Building on this, the MIME robot expanded therapeutic modalities by incorporating passive, active-assistive, and bilateral training modes, enhancing engagement in the rehabilitation process^3^. Further advancements are exemplified by the ARMin robot^4^, featuring an exoskeletal design with seven degrees of freedom, closely mimicking natural upper limb movements. This enables patients to perform complex tasks, fostering comprehensive motor recovery^5^. The Rice Wrist robot takes a different approach with its parallel mechanism that minimizes friction and backlash, ensuring precise control for wrist rehabilitation. This design is crucial for maintaining the natural alignment of the wrist during therapy, reducing the risk of discomfort or injury^6,7^. Continuing this trajectory, the Open Wrist robot offers further enhancements in efficiency, performance, and hand compatibility. Retaining the RRR mechanism of its predecessor, it introduces a linear degree of freedom to address misalignments between the user’s joint and the robot. Each degree of freedom is driven by a DC motor, with capstan cables ensuring smooth, backlash-free power transmission^8^. Also, the NU-Wrist robot represents a significant step in wearable rehabilitation robotics. Its exoskeletal structure can be either mounted on a table or worn by the patient, offering passive degrees of freedom to compensate for misalignments, thereby ensuring safety and comfort throughout the therapy session^9^. In^10^, a bionic cable-driven mechanism was developed for forearm-wrist rehabilitation, mimicking natural wrist movements with three degrees of freedom. This device features a spring within a parallel mechanism to counteract cable slack, ensuring smooth operation. Additionally^11^, presents a four-link parallel end-effector robot for wrist rehabilitation. This robot uses biomimetic muscle actuators for compliance and a fuzzy-based model to address the nonlinear behavior of the actuators. An adaptive controller, based on this model, guides the robot’s end-effector to follow and adjust trajectories, ensuring effective rehabilitation in various wrist stiffness regions^12^. The M3Rob is a 3-DoF wrist exoskeleton designed for rehabilitation. It features a force sensor, closed-loop admittance control, and can assist with both wrist and hand therapy. The device offers a wide range of motion and generates the necessary forces for daily activities, making it an effective tool for motor recovery^13^. Building on these advancements^14^, introduces a wrist rehabilitation robot utilizing a parallel four-bar mechanism for precise trajectory tracking and adaptive stiffness control. With two rotary actuators and a linear actuator, the robot supports comprehensive wrist movements, ensuring accurate and tailored assistance. The integration of position and force control allows for adaptive therapy, addressing individual stiffness variations effectively^15^. improves wrist rehabilitation robots by enhancing energy efficiency through a fuzzy adaptive algorithm. The optimized control strategy ensures precise energy management and boosts rehabilitation effectiveness. Beyond mechanical improvements, recent studies have also investigated the physiological effects of wrist rehabilitation robots. For instance, the influence of interactive force thresholds on muscle activation during isometric training has been analyzed, revealing how different force levels impact muscular endurance and recovery^14^. Additionally, advancements in control strategies have introduced real-time joint impedance estimation methods, allowing wrist exoskeletons to adaptively adjust training trajectories based on patients’ motion intention. These improvements enhance not only rehabilitation effectiveness but also interaction safety and user engagement^16^.

Recent studies have explored various aspects of wrist rehabilitation robots to enhance their effectiveness. In^17^, a lightweight and comfortable exoskeleton was designed with active and passive degrees of freedom to improve user adaptability. Further research in^18^ introduced advanced inverse kinematics analysis using artificial neural networks (ANN) and adaptive neuro-fuzzy inference systems (ANFIS) to enhance movement accuracy and control robustness. Additionally^19^, developed an optimized Takagi–Sugeno-Kang (TSK) fuzzy system, integrating hybrid Particle Swarm Optimization (PSO) and Genetic Algorithm (GA) to refine inverse kinematics performance. These advancements contribute to the continuous evolution of wrist rehabilitation robots, making them more precise, adaptive, and user-friendly.

Despite substantial progress in robotic rehabilitation, many systems still lack adaptability to evolving therapeutic demands. Conventional devices are typically built around fixed-degree-of-freedom configurations, limiting their ability to address diverse treatments; moreover, assembly and operation often require specialized expertise, hindering broader clinical adoption^20,21^. To address these challenges, modular robotic systems provide a compelling solution. These systems offer flexibility, adaptability, and ease of customization. Unlike conventional robots with rigid, predefined structures, modular robots can be easily reconfigured to meet different rehabilitation demands. The ability to swap modules quickly enhances user-friendliness and accessibility. Furthermore, modular designs allow for future upgrades, making it possible to add new features or improve functionality without needing a complete redesign^21^. In this vein, ref^22^. reports a low-cost modular manipulator for ADL with 4-DoF modules, an automated four-bar (crank–rocker) docking mechanism, and a suction end-effector, emphasizing printability, cost, and benchtop validation.

Recent studies have further demonstrated the growing importance of modularity in upper-limb rehabilitation robots. Modular exoskeletons have been developed to allow reconfigurable structures that can target different joints such as the shoulder, elbow, and wrist, thereby supporting both isolated and coordinated motion rehabilitation^22–25^. Such systems provide flexible combinations of modules to adapt to various anatomical and functional requirements of patients. In particular, soft and reconfigurable exoskeletons with modular joints have been shown to enhance compliance and comfort during training while simplifying maintenance and mechanical adjustment^24,25^.

At the component and subsystem level, modular architectures employing standardized actuator and link modules enable independent design, testing, and replacement of hardware units. These configurations facilitate simple scalability—for instance, extending a 4-DoF system to 7-DoF by adding identical modules—without the need for redesigning the entire robot^26^. Large-scale modular platforms have also been reported with combined mechanical and electronic modularity, featuring decentralized actuator-level control, multi-layered safety strategies, and highly flexible reconfiguration capabilities that allow operation as either compact 10-DoF systems or full-body 30-DoF rehabilitation setups^27^.

The integration of series-elastic and compliant actuation within modular designs has further improved user safety, motion smoothness, and human–robot interaction quality, as demonstrated in recent clinical-oriented systems^28,29^. Beyond conventional modularity, self-reconfigurable exoskeleton concepts have been proposed in which the robot can adapt its structure automatically in response to load distribution. These approaches employ finite-element analysis and graph-based algorithms to identify inactive components and reconfigure them toward high-stress regions—offering a visionary yet still largely experimental framework for intelligent structural adaptability^30^. Finally, optimization-oriented modular designs have highlighted how modularity simplifies both the physical construction and control system tuning processes, reinforcing its role as a key enabler of efficient and customizable rehabilitation systems^22^.

To address the high costs and structural complexities (e.g., weight and intricacy) commonly found in upper-limb rehabilitation robots, this paper adopts a modular design concept. The goal is to achieve a simplified overall robot architecture that avoids the complexities of more rigid models such as ANYexo, while still maintaining functional performance. This design aims to develop a modular upper-limb robotic system that can serve as a whole unit, offering portability or fixed support during Activities of Daily Living (ADL) for patient convenience.

In this study, we present the design, analysis, and development of a modular wrist rehabilitation robot that leverages the advantages of modular systems to create a more adaptable and patient-centered solution. The robot is equipped with three degrees of freedom for forearm and wrist movements. The modular structure of the robot is its most important feature, allowing for easy assembly and disassembly of the modules, making it possible to use a single module for rehabilitation if needed. This flexibility not only enhances ease of use but also provides high adaptability to various therapeutic demands. The modular design ensures that the robot can be customized based on the patient’s unique needs and recovery stage, allowing for a scalable and user-friendly approach to wrist rehabilitation. To improve control performance, an impedance control strategy was implemented along with a gravity compensation method to ensure smooth interaction between the user and the robot. The robot’s performance was validated through both simulation and experimental tests, confirming its effectiveness in wrist rehabilitation.

**Innovations of this research**:

- Modular design that enables the robot to be easily reconfigured for a variety of therapeutic tasks. Simplification in modular design makes it easier to achieve substantial cost-effective, light-weight structure, and ease of integration. Moreover, the presence of an inter-module docking system enables easy assembly and disassembly for rapid changes to different configurations. Homogeneous modules are designed to provide a distinct advantage wherein same modules can be used for rehabilitation of both upper limbs (bilaterally).
- Interchangeable modules that allow for rapid adjustments without requiring specialized technical knowledge, along with easy assembly and maintenance.
- Adaptability to different hand sizes, ensuring a personalized fit for various patients.
- Capability to support future upgrades, accommodating advancements in rehabilitation.

The structure of the paper is organized as follows: Sect. 2 presents the conceptual and detailed design of the robot. Section 3 analyzes the kinematics and dynamics of the system. Section 4 describes the control algorithm in detail. Section 5 reports the simulation and experimental results. Finally, Sect. 6 concludes the paper with a summary of the findings.

## Mechanical design

### Design requirements and conceptual design

**Fig. 1:**
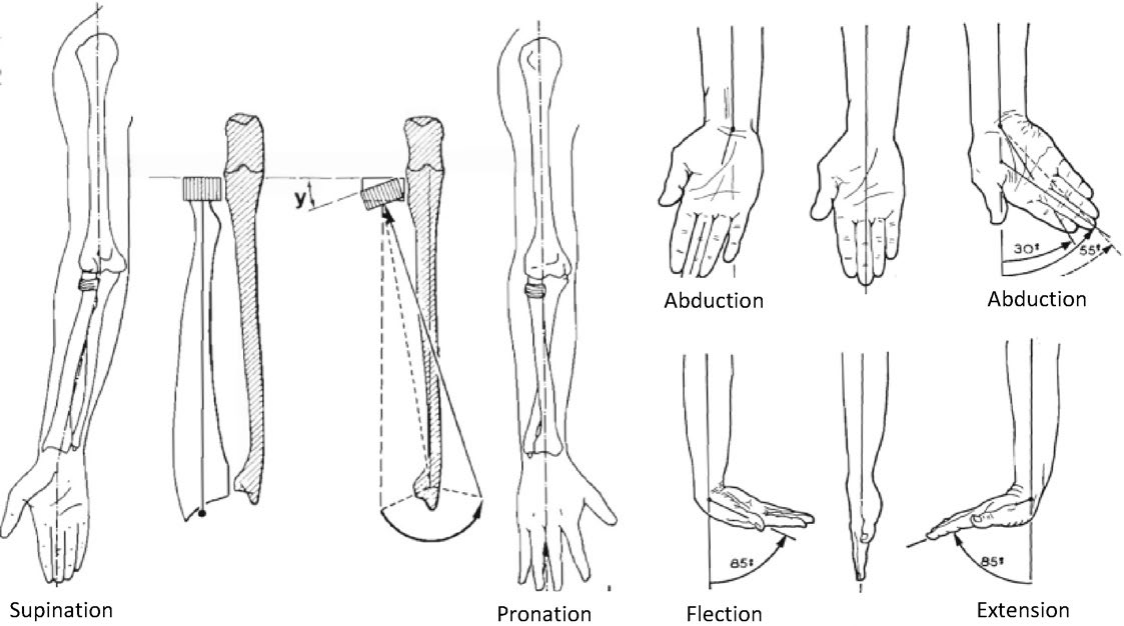
Human wrist degrees of freedom^32^.

Attention to general design requirements is a crucial aspect of the design process. In the design of a wrist rehabilitation robot, a critical consideration is ensuring that the robot’s joints align with the natural movements of the human wrist. For optimal functionality, the robot’s joints must be highly compatible with the wrist’s natural motion, ensuring precise alignment between the robot’s rotational axes and those of the human wrist. Any misalignment could result in unintended forces and torques, potentially causing harm to the user’s wrist^31^. The human wrist, along with the forearm, has three degrees of freedom. Pronation/Supination (PS) is associated with the forearm, while two movements Flexion/Extension (FE) and Abduction/Adduction (AA) are related to the wrist, as shown in Fig. 1^9,32^. To replicate these movements, the design of the robot should incorporate three orthogonal rotational joints, providing three degrees of freedom. However, in practice, the AA axis is slightly offset by approximately 5 mm at a small angle relative to the FE axis, making perfect alignment across different individuals challenging^33^. To address this, the design must include two passive degrees of freedom at the end effector, particularly along the Abduction/Adduction direction, allowing for free wrist movement and minimizing unwanted forces. Another crucial aspect is the wrist’s range of motion. The robot must cover the full range of motion necessary for daily tasks while ensuring that the wrist does not exceed its natural limits to avoid discomfort or injury. As shown in Table 1, both the human wrist’s range of motion and the range considered for the robot’s design should be carefully taken into account to ensure functionality and safety^31^. Additionally, the robot must allow users to adjust the settings based on their wrist size, preventing excessive torque and force on the wrist. To facilitate this, the robot’s links should feature a telescopic structure, enabling users to adjust the link length according to their hand size. Furthermore, the robot’s design should prioritize user comfort and ease of use, considering factors such as size, shape, weight, and access to various functionalities. Since the wrist rehabilitation robot must support diverse capabilities, users should be able to customize the robot’s mechanism according to their needs and therapeutic requirements. This customization should allow easy modifications to the robot’s settings. Given these requirements, the objective is to design a modular robot. Modular robotics offers distinct advantages over other robotic technologies, including flexibility in configuration and ease of construction and assembly.

**Table 1:** Range of motion of the human wrist and the designed robot.

Movement	Human wrist range of motion (deg)^32^	Robot’s considered range (deg)
Supination/pronation	$\:90/85$	$\:80$
Flexion/extension	$\:85/85$	$\:50$
Abduction/adduction	$\:25/45$	$\:20$

### Conceptual design

**Fig. 2:**
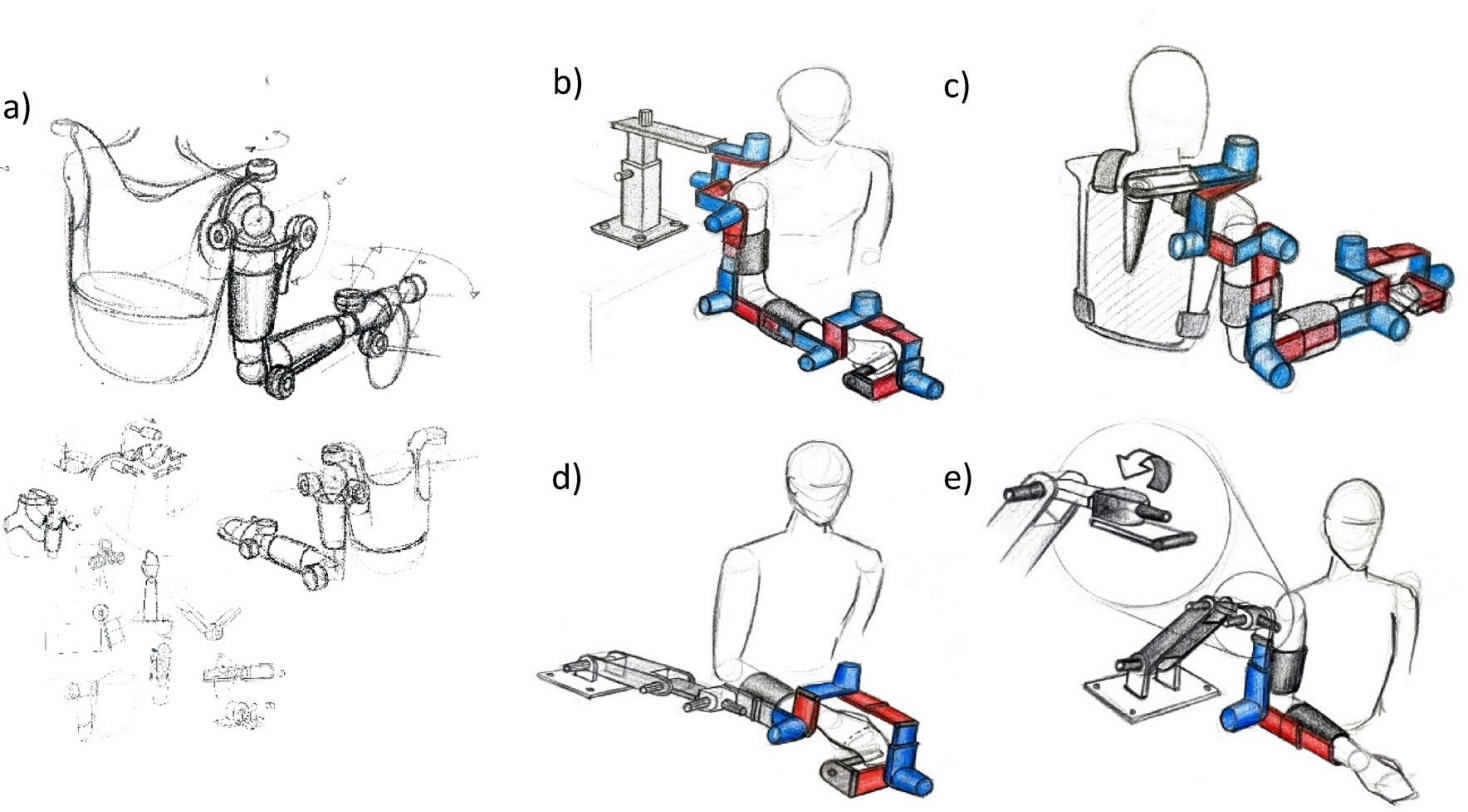
Overview of conceptual desgin process.

**Fig. 3:**
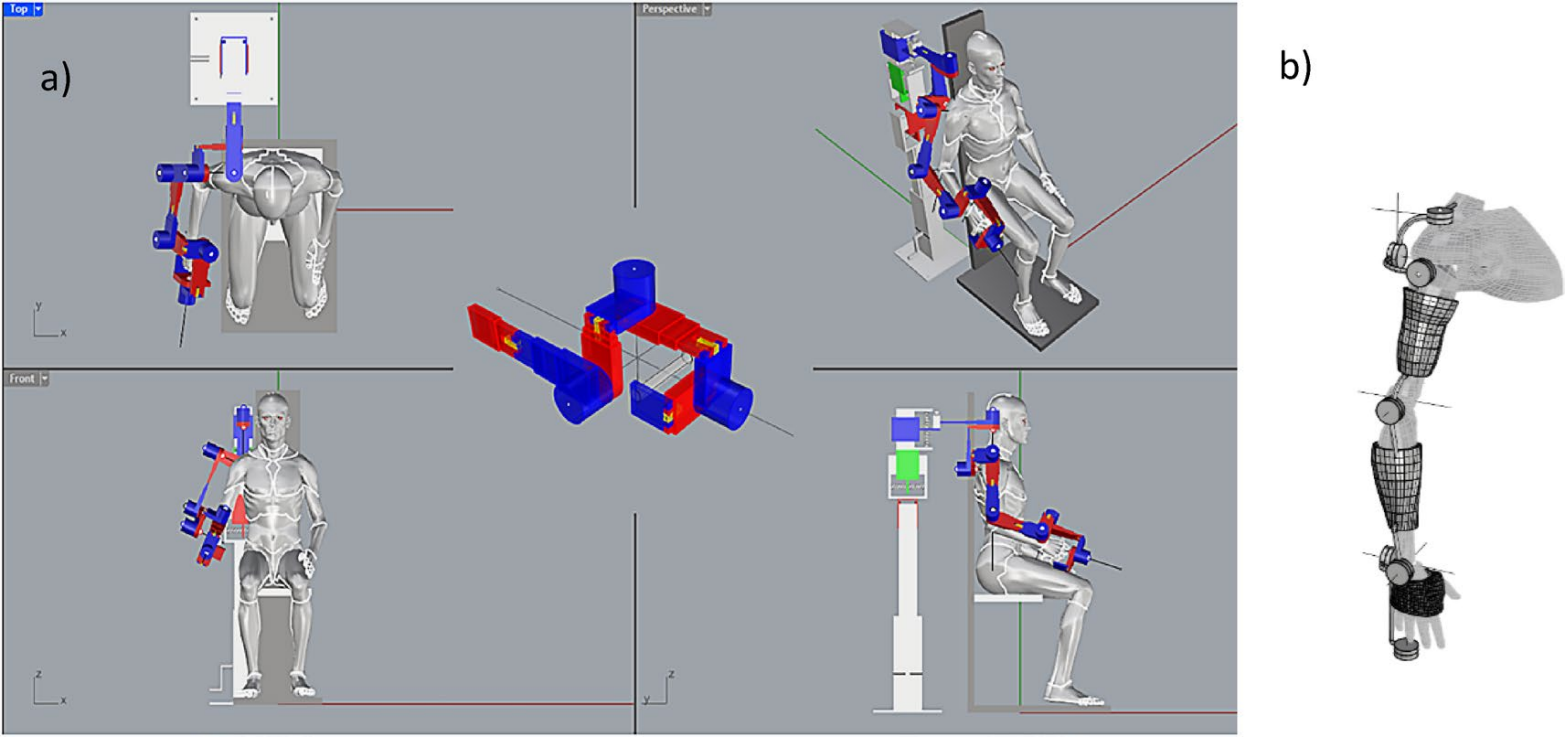
Conceptual desgin in rhino.

Figures 2 and 3 illustrates the complete process of developing the design concept, from the initial sketch to the final model created in Rhino software. In Fig. 2a, a preliminary schematic of the overall design is presented, where the modules are uniform, enabling straightforward installation and configuration. This modular design accommodates both the elbow and shoulder joints. Figure 2b and c depict the schematic design of the upper limb with fixed and movable supports, respectively. Considering cost constraints and the principle of minimizing complexity in modular robot design, we opted for the fixed support for further development. Figure 2d and c showcase the conceptual design configuration for the wrist and forearm. Figure 3a provides an overview of Fig. 2a, allowing for the evaluation of anthropometric parameters in Rhino software. Finally, Fig. 3b presents a comprehensive perspective of the robot and its integration with the hand.

Next, we will discuss the customization of this design, focusing on the details of the hand assembly. Since the primary objective of this article is to analyze the wrist, the design of this section is still in progress. The wrist module is designed with constrained revolute degrees of freedom, consisting of three modules, ultimately forming an RRR serial configuration.

### Detail design of the robot

To design a modular robot effectively, the links must accommodate different joint degrees of freedom and allow adjustable length. To achieve this flexibility, a telescopic structure was adopted for the links. Each module consists of two links that rotate relative to each other, forming a revolute joint. A Dynamixel servo motor is mounted on one link and connected to the adjacent link through a short output steel shaft, enabling controlled rotation. The revolute connection between the two links is realized by directly coupling the actuator’s output shaft to the distal link through a shaft–link coupler. Each link itself comprises three nested sliding segments that move telescopically, allowing length adjustment according to the user’s forearm size.

**Fig. 4:**
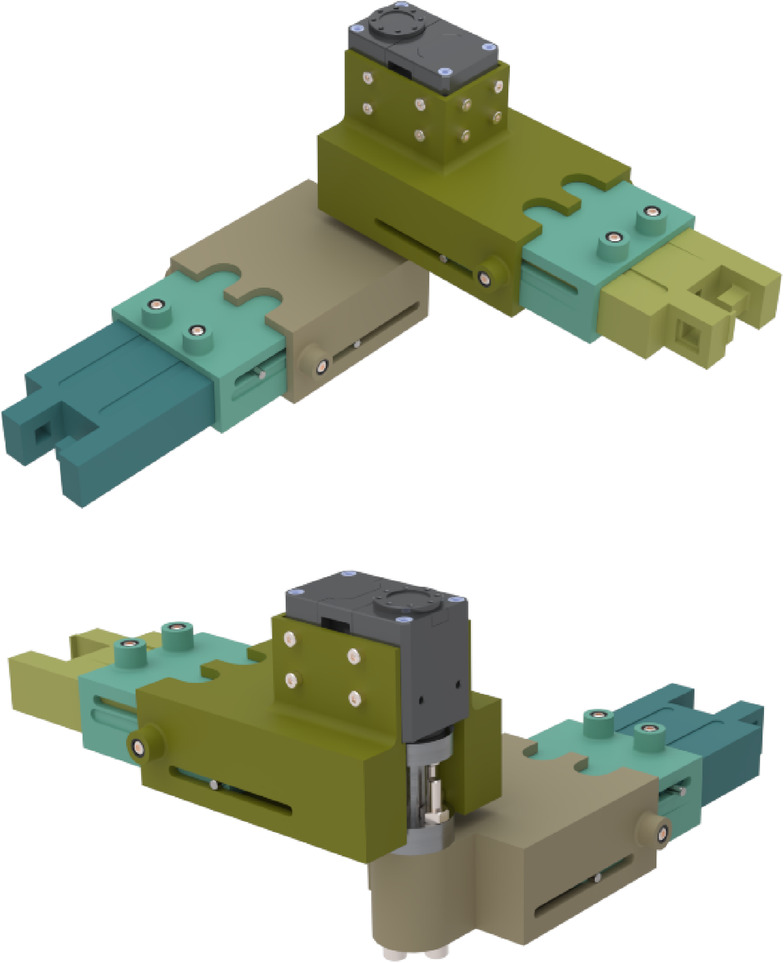
3D view of a module of robot.

In this configuration, radial and axial reaction loads generated at the joint are primarily supported by the internal bearings of the Dynamixel servo, which are designed to carry such loads for light- to medium-duty applications. The driving torque is transmitted through the shaft–link coupler to the following link. Since this study focuses on the wrist joint, where the experienced torques and reaction forces are considerably lower than those at the elbow or shoulder, the internal bearing capacity of the selected servo is sufficient. In future high-load applications, however, an external angular-contact bearing can be integrated into the joint housing to further increase radial and axial load capacity and extend service life. Figure 4 illustrates a 3D view of a robot module and its telescopic structure.

**Fig. 5:**
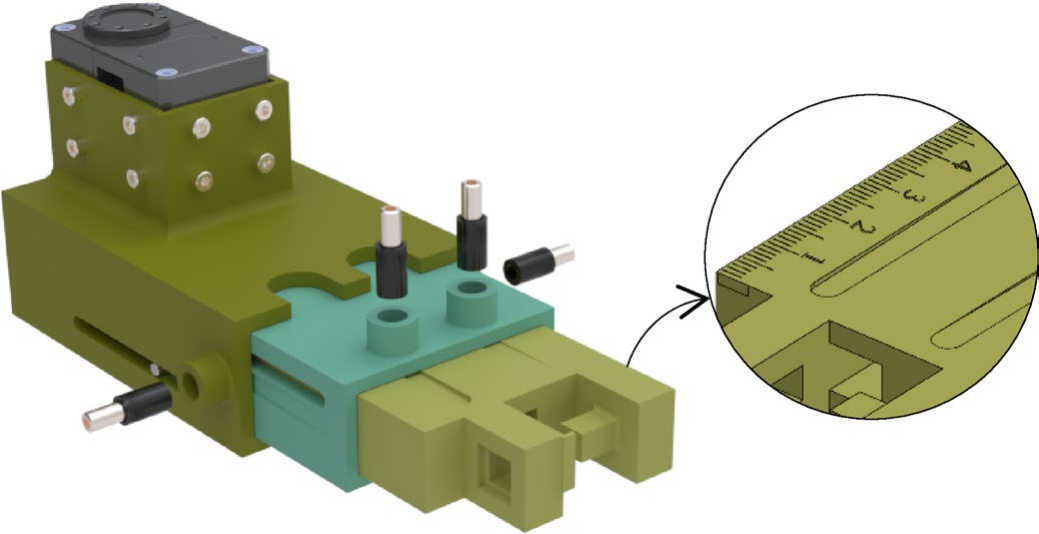
Fixing the distance between components (exploded view).

As shown in Fig. 5, the distance between the two pieces is adjusted using two set screws positioned inside a rivet nut. Tightening these screws clamps the nested parts and prevents sliding. Furthermore, there is a pinned guide along the first and second parts of each link that ensures smooth linear motion while suppressing unwanted rotation. Another feature is the presence of two sloped (tapered) slots in the moving element; after tightening the set screws, these slots create a wedging effect that resists unintended motion during operation.

The revolute connection between the two links is realized by coupling the actuator’s output steel shaft to the distal link. In this prototype, the selected actuator is a Dynamixel servo, whose all-in-one design (DC motor, gear train, sensor, controller) provides a compact joint module. Radial and axial reaction loads are primarily carried by the servo’s internal bearings supporting its short shaft, while the driving torque is transmitted through the shaft–link coupler. Since this study targets the wrist joint—where the expected forces/torques are much lower than at the elbow or shoulder—the selected servo is sufficient to withstand the anticipated radial and axial loads. If higher loads are required in future configurations, an external angular-contact bearing can be added on the housing to augment radial/axial capacity.

**Fig. 6:**
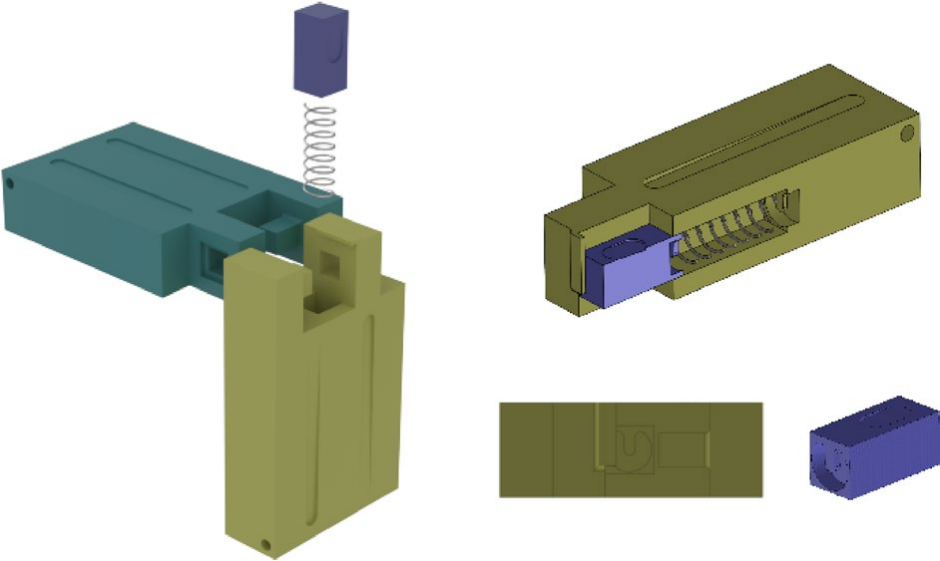
Two module connection design.

**Fig. 7:**
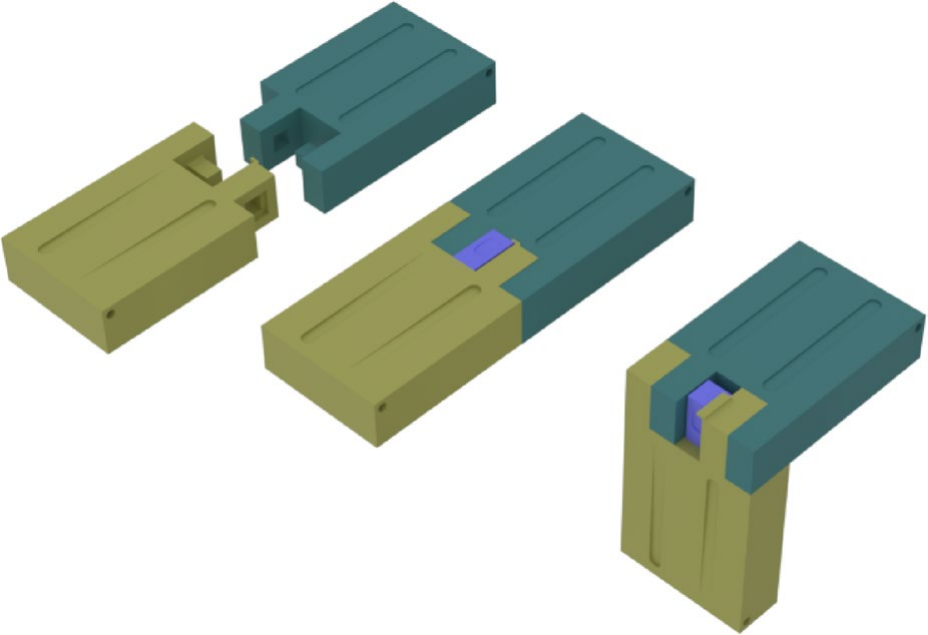
Vertical and horizontal connection of two modules.

To connect modules, the end of each link is designed so the two pieces can manually detach. Figure 6 shows an exploded view of two pieces from adjacent modules connected vertically. In this design, the yellow part provides a receptacle for the purple piece; pressing the purple piece downward disengages the latch. A pair of hooks on the purple and yellow pieces retains a spring, and a matching protrusion ensures positive seating inside the cylindrical pocket, preventing accidental release. The same interface allows modules to be attached vertically or horizontally, enabling versatile assembly options (Fig. 7).

**Fig. 8:**
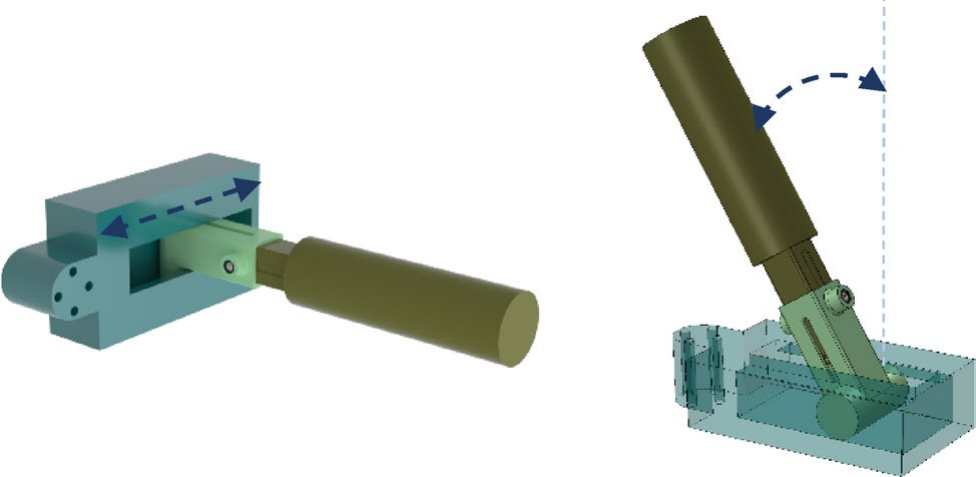
End-effector of the robot.

The configuration of the robot plays a critical role in the design of the end-effector, as it determines whether the end-effector can be oriented vertically or horizontally, depending on the specific application. A crucial consideration in this design is addressing the displacement of the abduction/adduction axis of motion. This becomes particularly important because the abduction/adduction and flexion/extension axes of the wrist joint are neither orthogonal nor intersecting. These axes are separated by a small, yet significant, distance of approximately 5 mm, which can vary slightly depending on the type and range of movement^33^. Consequently, it is essential that the robot’s design accommodates this displacement to maintain proper alignment and minimize any potential misalignment or unintended strain during rehabilitation. As shown in Fig. 8, the system incorporates two passive degrees of freedom: one for the linear displacement of the axis and another for its rotational movement. This approach ensures that additional forces are not unintentionally applied to the wrist, thereby enhancing the rehabilitation process.

**Fig. 9:**
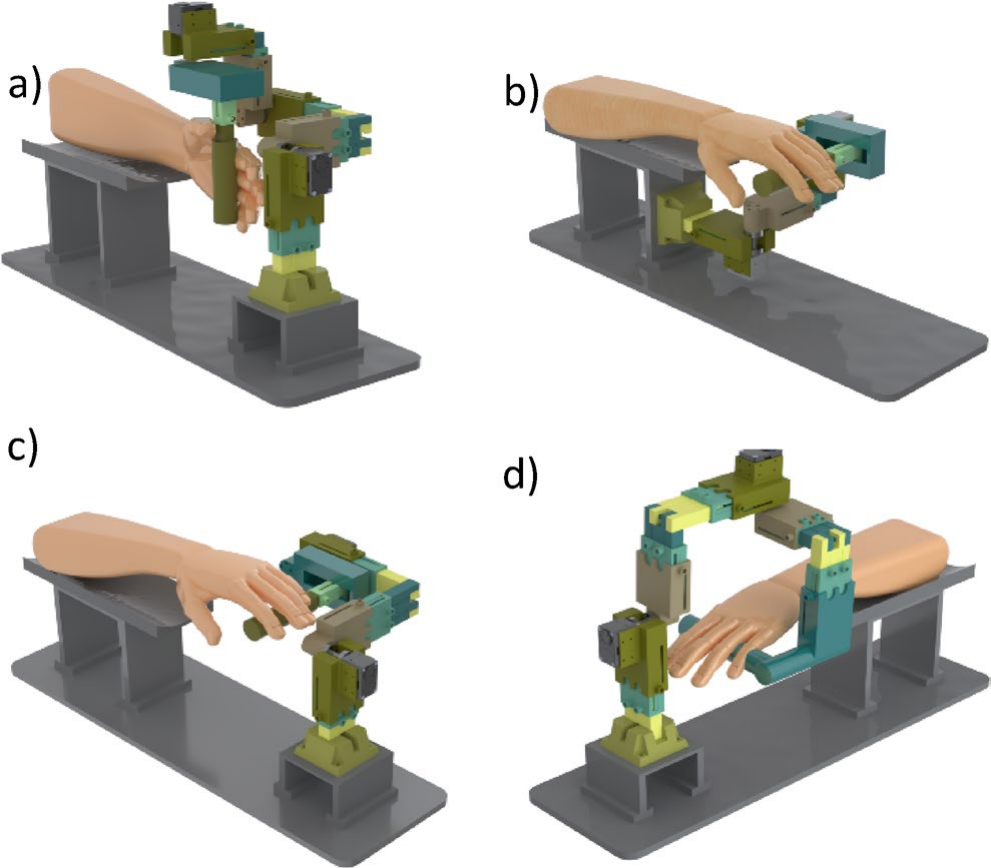
Configurations for different degrees of freedom: (**a**) PS/AA/FE, (**b**) AA/FE, (**c**) PS/FE, (**d**) PS/AA.

As mentioned, one of the key features of a modular robot is its adaptability to different tasks. In other words, a modular robot should be designed in a way that allows it to be assembled into various configurations with different degrees of freedom. In Fig. 9, different configurations for a three-degree-of-freedom and a two-degree-of-freedom robot are presented.

### Sequence of DOF selection

**Fig. 10:**
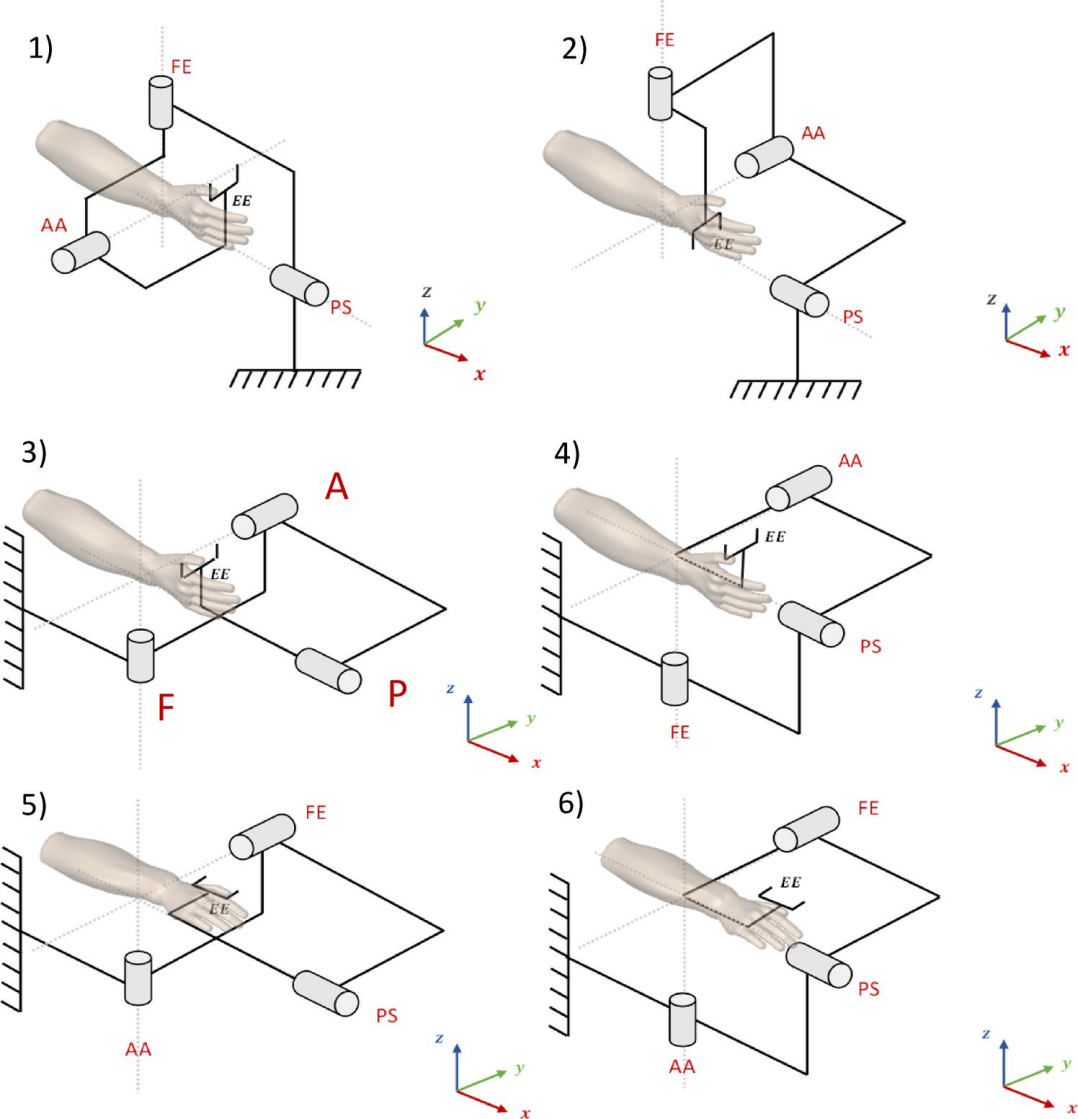
Different configuration of 3-DOF modular robot.

The accurate selection of the order of revolute joints has been identified as a key factor in the design of a modular robot. The degrees of freedom (DOF) have been chosen to ensure that the motors generate the required torque for both hand and robot movement. Additionally, it has been verified that the forces applied by the device do not exceed the motors’ operational limits. As shown in Fig. 10, six possible configurations have been considered, and an analysis has been conducted to determine the most suitable one.

Initially, a design assuming three degrees of freedom for the robot was developed and subsequently simulated in Simscape, considering various configurations of degrees of freedom. The required torque for each motor’s movement, along with the radial and axial forces acting on each motor, were obtained as critical parameters for selecting an appropriate configuration. However, since the torque on the motors varies at different movement angles, the maximum torque for different actuator angles was calculated. To determine the required torque for the first motor, a sinusoidal motion with a maximum angular velocity of π/2 (rad/sec)^34^ and the corresponding domain was defined. The subsequent motors were positioned at different locations, and the maximum torque was calculated for each case. This procedure was replicated for the other motors. In addition to the device’s mass, a mass of 600 g was considered to represent the user’s hand.

**Fig. 11:**
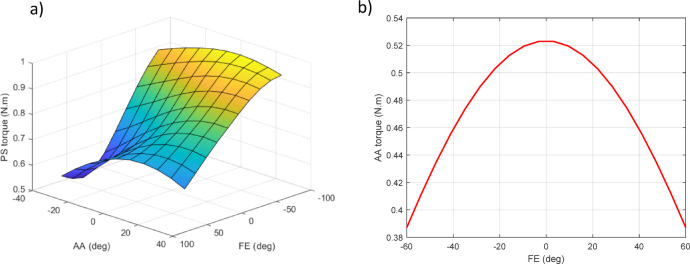
PS_AA_FE configuration. (**a**) Required torque for the first motor. (**b**) Required torque for the second motor.

For example, in the PS-AA-FE configuration (as shown in Fig. 10.1), the maximum required torque for the first motor, as shown in Fig. 11a, was calculated for different positions of the second and third motors. The maximum torque for the sinusoidal abduction/adduction axis was evaluated under different positional configurations of the third motor, as illustrated in Fig. 11b.

**Fig. 12:**
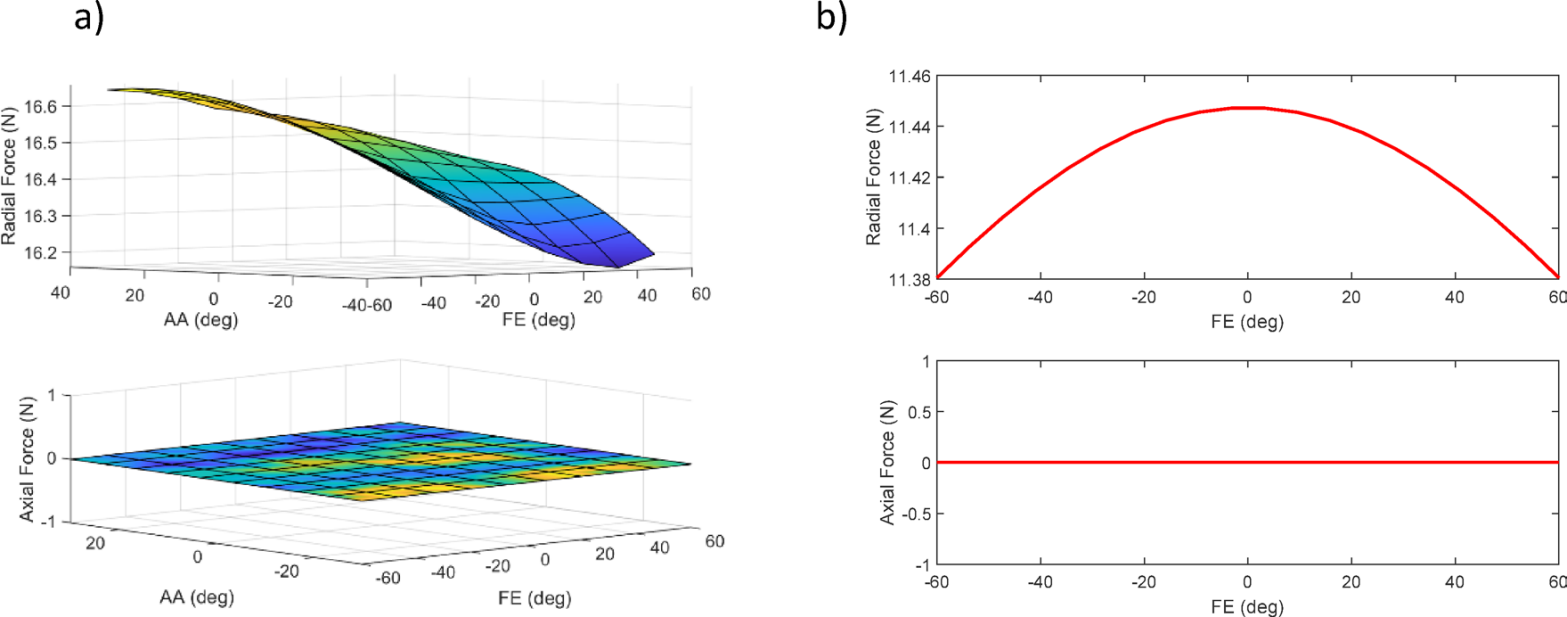
PS_AA_FE configuration. (**a**) Forces on the first motor. (**b**) Forces on the second motor.

Another important parameter to consider when determining the order of degrees of freedom is the calculation of radial and axial forces on the motor shaft. Since motor specifications define allowable limits for these forces, an improper configuration may lead to motor damage. Figure 12a illustrates the radial and axial forces acting on the first motor for different positions of the second and third motors, while Fig. 12b presents the radial and axial forces on the second motor for various positions of the third motor.

Five additional configurations of the robot were thoroughly examined in the same way, and the results are summarized in Table 2.

**Table 2:** Results of 6 configurations for three degrees of freedom.

Configuration	Actuator								
	Required motor torque (*N*.m)			Radial load acting on motor (*N*)			Axial load acting on motor (*N*)		
	First motor	Second motor	Third motor	First motor	Second motor	Third motor	First motor	Second motor	Third motor
PS-AA-FE	0.993	0.549	0.032	16.711	11.501	0.310	0	0	6.703
PS-FE-AA	0.988	0.0858	0.295	16.809	0.567	6.838	0	11.193	0
FE-AA-PS	0.1881	0.727	0.09	1.060	11.523	6.703	15.683	0	0
FE-PS-AA	0.286	0.620	0.236	1.439	11.640	6.811	15.683	0	0
AA-FE-PS	0.093	0.731	0.009	0.709	11.595	6.703	15.683	0	0
AA-PS-FE	0.144	0.658	0.236	0.719	11.709	6.833	15.683	0	0

There are limitations in the construction of the fourth and sixth configurations (Fig. 10.4 and Fig. 10.6) due to the wide range of motion (ROM) in supination/pronation, which causes link and end-effector collisions. Configurations should also be selected to minimize the axial force encountered by the motors, as the maximum axial force that motors can typically withstand is lower than the maximum radial force. Consequently, the robot’s weight is axially supported by the first motor, making configurations from the third to the sixth less suitable. Therefore, a configuration that allows the motor to support the robot’s weight radially is recommended. Both the first and second configurations (PS-AA-FE and PS-FE-AA) are viable options, with the first configuration being the preferred choice. This configuration limits movement, reducing the need to lengthen the connections to avoid collisions, thus placing the robot in its safest operating condition and decreasing the torque applied to the motors. Based on the obtained results, the selected motors must provide at least 2 N·m of torque. It is important to note that these results are based on the simulation of an initial robot model. In practical applications, the robot’s weight may fluctuate, or it may operate under the control of different individuals. Additionally, the actuator–bearing assembly must be rated to withstand at least 15 N of radial load.

## Kinematic and dynamic of the robot

### Kinematic modeling

The robot’s kinematic model is inspired by the anatomy of the human wrist. The mechanism is modeled with three rotational degrees of freedom—pronation/supination (PS), abduction/adduction (AA), and flexion/extension (FE)—that intersect at an approximately common anatomical pivot. The Denavit–Hartenberg (DH) convention is used to derive the kinematic equations^35^. We define the base frame at the wrist joint, i.e., at the nearly concurrent intersection of these three axes. This choice is motivated by both biomechanical and kinematic considerations. Biomechanically, the three axes pass through approximately the same anatomical region of the wrist, forming a common pivot about which the hand primarily rotates; consequently, their combined motion predominantly affects the orientation of the end-effector, while the position of the wrist center remains nearly constant. Kinematically, placing the base frame at this intersection simplifies the transformation matrices because the first link frame coincides with the physical center of rotation of the wrist. It also yields a more intuitive and explicit representation of the end-effector workspace, since any displacement observed at the gripper originates from rotations about this anatomical reference rather than from compounded link translations.

**Fig. 13:**
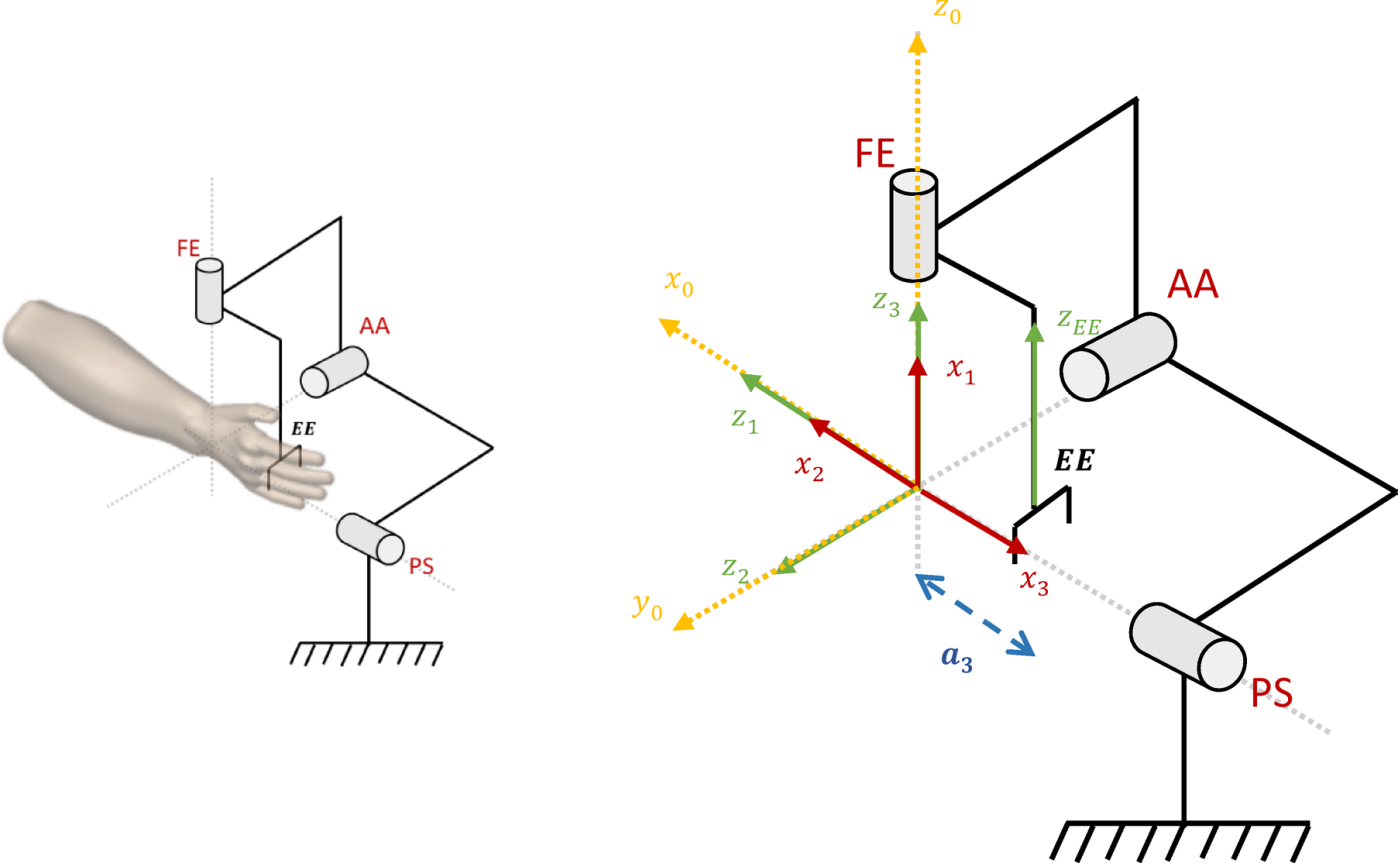
Kinematics model of wrist rehabilitation robot.

Placing the base frame elsewhere would introduce unnecessary translational offsets, obscure the actual workspace geometry, and complicate the forward and inverse kinematic derivations. Therefore, locating the base frame at the wrist joint provides a more faithful anatomical correspondence and a mathematically simplified motion model. The end-effector frame is located at the robot’s gripper. Figure 13 illustrates the kinematic parameters; the corresponding values are listed in Table 3.

**Fig. 14:**
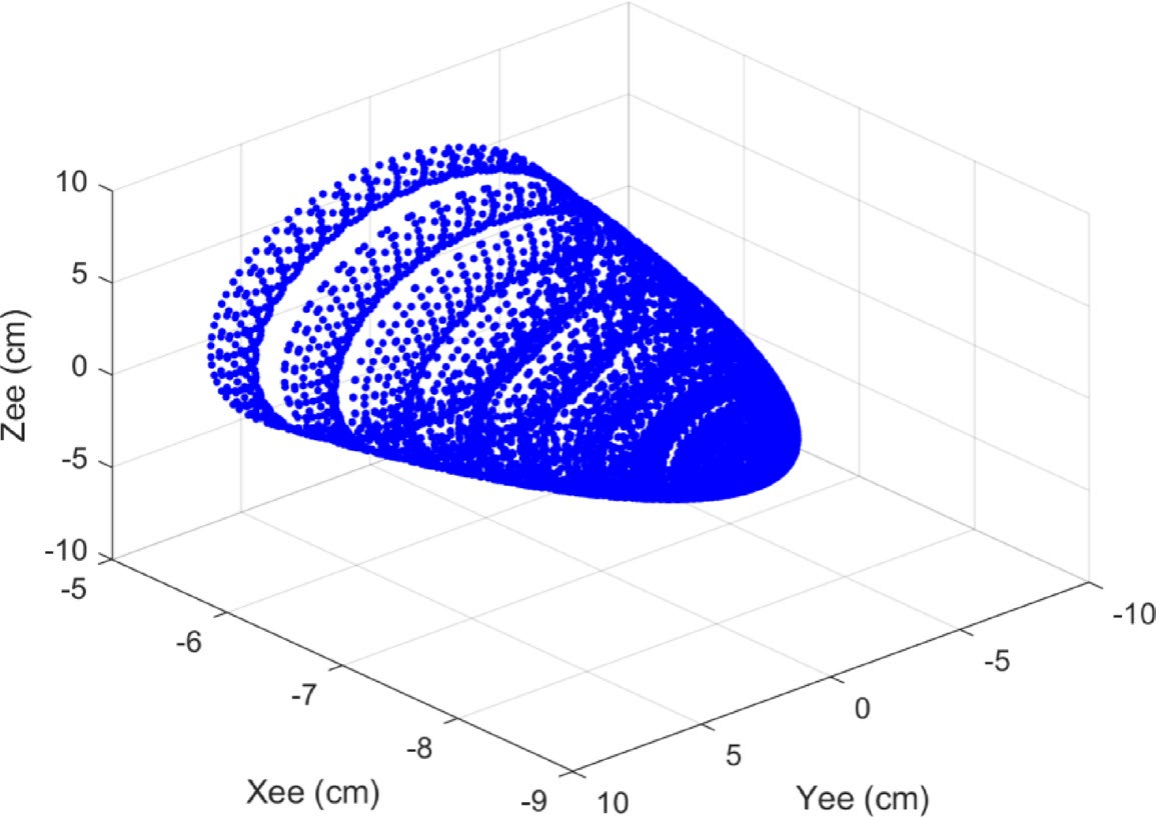
The workspace of the robot’s end-effector.

In this robot, $\:{a}_{3}$ represents the distance from the wrist to the end-effector, which is considered to be 8.5 cm. Using the transformation matrix $\:{}^{i-1}{T}_{i}$ (Eq. (1)), the transformation matrices for each coordinate frame are calculated. Finally, the position of the end-effector relative to the base frame is obtained. The robot’s workspace is shown in Fig. 14.

1 $${}_{}^{i - 1}{T_i} = \left[ \begin{array}{llll} {\cos \left( {{\theta _i}} \right)} & { - \sin \left( {{\theta _i}} \right)} & 0 & {{a_{i - 1}}} \\ {\sin \left( {{\theta _i}} \right)\cos \left( {{\alpha _{i - 1}}} \right)} & {\cos \left( {{\theta _i}} \right)\cos \left( {{\alpha _{i - 1}}} \right)} & { - \sin \left( {{\alpha _{i - 1}}} \right)} & { - \sin \left( {{\alpha _{i - 1}}} \right){d_i}} \\ {\sin \left( {{\theta _i}} \right)\sin \left( {{\alpha _{i - 1}}} \right)} & {\cos \left( {{\theta _i}} \right)\sin \left( {{\alpha _{i - 1}}} \right)} & {\cos \left( {{\alpha _{i - 1}}} \right)} & {\cos \left( {{\alpha _{i - 1}}} \right){d_i}} \\ 0 & 0 & 0 & 1 \\ \end{array} \right]$$

**Table 3:** The DH parameters of wrist rehabilitation robot.

$\:{i}$	$\:{{\alpha\:}}_{{i}-1}\left({d}{e}{g}\right)$	$\:{{a}}_{{i}-1}\left({m}{m}\right)$	$\:{{d}}_{{i}}\left({m}{m}\right)$	$\:{{\theta\:}}_{{i}}\left({d}{e}{g}\right)$
$\:1$	$\:-90$	$\:0$	$\:0$	$\:-90+{\theta\:}_{1}$
$\:2$	$\:90$	$\:0$	$\:0$	$\:90+{\theta\:}_{2}$
$\:3$	$\:90$	$\:0$	$\:0$	$\:180+{\theta\:}_{3}$
$\:{E}{E}$	$\:0$	$\:{a}_{3}$	$\:0$	$\:0$

### Dynamic modeling

The joint torques of the robot are computed using the Newton-Euler method, following the algorithm proposed by Luh, Walker, and Paul^36^. This method consists of two main steps: first, the velocities and accelerations of the links are recursively computed from the base to the $\:{n}_{th}$ link. Then, by applying Newton-Euler equations, the forces and torques acting on each link are obtained. The Newton equation, shown in Eq. (2), relates the applied force to the acceleration of the link’s center of mass. Similarly, the Euler equation, given in Eq. (3), describes the torque at the center of mass of the $\:{i+1}_{th}$ link based on its angular acceleration and inertia properties.

2 $$\:{}^{i+1}{F}_{i+1}={m}_{i+1}{}^{i+1}{\dot{{V}_{c}}}_{i+1}$$

3 $$\:{}^{i+1}{N}_{i+1}={}^{{C}_{i+1}}{I}_{i+1}{}^{i+1}{\dot{\omega\:}}_{i+1}+{}^{i+1}{\omega\:}_{i+1}\times\:{}^{{C}_{i+1}}{I}_{i+1}{}^{i+1}{\omega\:}_{i+1}$$

Once these forces and torques are determined, the equilibrium equations are applied to compute the joint torques. By summing the forces acting on link $\:i$, the force equilibrium equation is given by Eq. (4), and the torque equilibrium is expressed by Eq. (5), In these equations, $\:{}^{i}{f}_{i}$ and $\:{}^{i}{n}_{i}$ represent the force and torque vectors acting on link $\:i$, expressed in the local coordinate frame $\:i$, resulting from the interaction between link $\:i$ and link $i-1$. $\:{}^{i}{F}_{i}$ and $\:{}^{i}{N}_{i}$ denote the external force and external torque applied to link *i* (such as gravity or contact effects). $\:{}^{i}{R}_{i+1}$ is the rotation matrix that transforms a vector from frame $\:i+1$ to frame $\:i$. $\:{}^{i}{P}_{i+1}$ defines the position vector from the origin of frame $\:i$ to the origin of frame $\:i+1$, while $\:{}^{i}{{P}_{c}}_{i}$ indicates the position vector from the origin of frame $\:i$ to the center of mass of link $\:i$.

4 $$\:{}^{i}{f}_{i}={}^{i}{R}_{i+1}\:{}^{i+1}{f}_{i+1}+\:{}^{i+1}{F}_{i+1}$$

5 $$\:{}^{i}{n}_{i}={}^{i}{N}_{i}+{}^{i}{R}_{i+1}\:{}^{i+1}{n}_{i+1}+\:{}^{i}{{P}_{c}}_{i}\times\:{}^{i}{F}_{i}+{}^{i}{P}_{i+1}\times\:{}^{i}{R}_{i+1}\:{}^{i+1}{f}_{i+1}$$

Finally, the joint torque $\:{\tau\:}_{i}$ is obtained by extracting the $\:Z\:$ component of the torque vector, as expressed in Eq. (6).

6 $$\:{\tau\:}_{i}={{}^{i}{n}_{i}}^{T}{}^{i}{\widehat{Z}}_{i}$$

Finally, the robot’s overall dynamic equation is rewritten as Eq. (7). In this equation, $\:M\left(\theta\:\right)$ represents the mass matrix, $\:V\left(\theta\:,\dot{\theta\:}\right)$ denotes the velocity matrix, and $\:G\left(\theta\:\right)$ corresponds to the gravity matrix.

7 $$\:M\left(\theta\:\right)\ddot{\theta\:}+V\left(\theta\:,\dot{\theta\:}\right)+G\left(\theta\:\right)=\tau\:$$

## Impedance control

### Principles of impedance control

Impedance control is a dynamic strategy that integrates force and position control, ensuring a balanced interaction between the robot and its environment^37^. This approach is particularly significant in rehabilitation robotics, where the robot must assist human movements safely and effectively without exerting harmful forces. Mechanically, impedance represents the system’s resistance to external motion. By controlling mechanical impedance, the robot regulates the reactive forces generated in response to external interactions. This is achieved by adjusting parameters such as stiffness, damping, and inertia. For instance, a robot with low stiffness can adapt flexibly to external forces, enabling smooth and compliant interactions. Conversely, high stiffness settings require significant force to induce movement, thereby providing stability. Impedance control offers significant advantages over pure position control or pure force control. In position control, the robot is rigidly driven to a predetermined location, potentially applying excessive forces if external constraints exist. In pure force control, effective regulation of the interaction force requires direct physical contact with the environment; this is not a drawback, but an inherent operational requirement of force control.

Impedance control, however, allows for an adaptive interaction, where both force and position are managed indirectly, minimizing the risk of applying harmful forces to the patient. Conceptually, impedance control can be regarded as a subcategory of force control; here, we use it to bridge the practical gap between pure position and pure force control.

This control strategy is particularly crucial for wrist rehabilitation robots, where precise control of both movement and applied forces is essential for patient safety and therapeutic success^38^. The relationship between force $\:F$ and velocity $\:V$ in impedance control is expressed as Eq. (8).

8 $$\:F=Z.V$$

Where $\:Z$ represents the mechanical impedance and $\:V$ is the velocity, which is the derivative of the relative displacement. The relative displacement $\:{X}_{r}$ is defined as the difference between the desired position $\:{X}_{d}$ and the actual position $\:X$ expressed as $\:{X}_{r}={X}_{d}-X$. In the Laplace domain, impedance is defined as Eq. (9).

9 $$\:Z\left(s\right)=\frac{F\left(s\right)}{s{X}_{r}\left(s\right)}\Rightarrow\:F\left(s\right)=sZ\left(s\right).{X}_{r}\left(s\right)$$

The desired impedance $\:Z\left(s\right)$ is often modeled as Eq. (10).

10 $$\:Z\left(s\right)={M}_{d}s+{C}_{d}+\frac{{K}_{d}}{s}$$

Where $\:{M}_{d}$, $\:{C}_{d}$and $\:{K}_{d}$ are the desired inertia, damping, and stiffness coefficients, respectively. By substituting $\:Z\left(s\right)$ into the Eq. (9), we get Eq. (11).

11 $$\:F\left(s\right)=\left({M}_{d}{s}^{2}+{C}_{d}s+{K}_{d}\right)\left({X}_{d}\left(s\right)-X\left(s\right)\right)$$

Taking the inverse Laplace transform, the force in the time domain is Eq. (12).

12 $$\:F\left(t\right)={M}_{d}\left(\ddot{x}-{\ddot{x}}_{d}\right)+{C}_{d}\left(\dot{x}-{\dot{x}}_{d}\right)+{K}_{d}\left(x-{x}_{d}\right)$$

### Impedance controller structure

As discussed, impedance control manages the interaction between the robot’s end-effector and the environment. In Eq. (12), the interaction force is determined by setting the coefficients $\:{M}_{d}$, $\:{C}_{d}$, and $\:{K}_{d}$. To calculate the desired joint torques, this force is multiplied by the transpose of the robot’s Jacobian matrix. In this study, only stiffness and damping are controlled, with the inertia coefficient neglected.

13 $$\:{\tau\:}_{3\times\:1}={{J}^{T}}_{3\times\:3}{{F}_{EE}}_{3\times\:1}$$

**Fig. 15:**
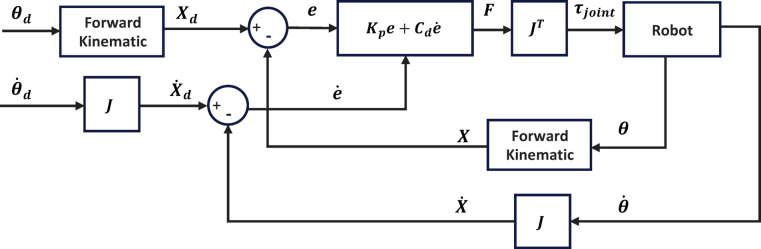
Block diagram of the impedance controller with path planning in joint space.

In Eq. (13), $\:\tau\:$ denotes the joint torques, $\:J$ represents the Jacobian matrix of the robot, and $\:{F}_{EE}$ is the force applied to the end-effector. Due to the absence of position and velocity sensors on the end-effector, feedback is obtained from the joint sensors. The position and velocity of the end-effector are subsequently computed by applying the forward kinematics and Jacobian matrices to the joint parameters. In wrist rehabilitation, where each degree of freedom corresponds to specific movements, it is preferable to plan the trajectory in joint space, as depicted in Fig. 15, and then map it to the end-effector space. This ensures precise control of joint movements, facilitating effective rehabilitation.

### Weight compensation

**Fig. 16:**
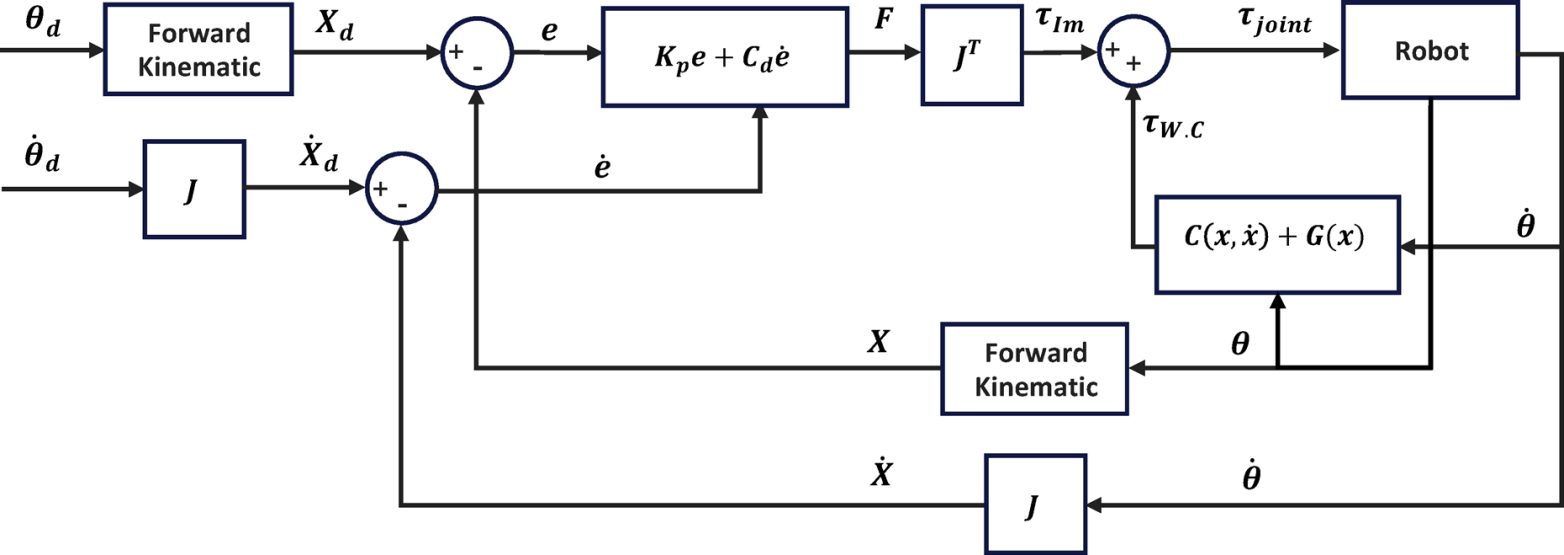
Block diagram of the impedance controller and weight compensator with path planning in joint space.

Weight compensation is essential in robotic systems, especially in rehabilitation robots, to reduce the impact of the robot’s weight on performance and motion accuracy and to ensure that the user does not perceive the device’s weight during operation. In our implementation, weight compensation is extended to dynamic feedforward compensation to eliminate the influence of the robot’s own dynamics on tracking accuracy and the user’s perceived effort. The feedforward terms are derived from the complete dynamic model described in Section ‎4.1. and include both the Coriolis/centrifugal term $\:C(x,\dot{x})$ and the gravity term $\:G\left(x\right)$. These terms are evaluated as functions of the robot’s joint configuration and velocity and applied directly to the actuators as feedforward torques. By compensating for these nonlinear effects, the impedance controller is relieved from counteracting the robot’s intrinsic dynamics and can focus solely on shaping the desired interactive behavior between the robot and the user. As shown in Fig. 16, the control structure combines impedance control with dynamic feedforward compensation. The impedance law $\:{K}_{p}\left(e\right)+{C}_{d}\left(\dot{e}\right)$ defines the desired force–motion relationship in task space, while the Jacobian transpose $\:{J}^{T}$ maps these interaction wrenches into joint torques. The overall control command is therefore $\:\tau\:={J}^{T}\left({K}_{p}\left(e\right)+{C}_{d}\left(\dot{e}\right)\right)+C\left(x,\dot{x}\right)+G\left(x\right)$ where the first term regulates interaction forces and the latter two compensate for the robot’s dynamic and gravitational effects. This integrated approach improves transparency (the user does not feel the robot’s weight) and stability, yielding smooth and natural assistance during rehabilitation exercises.

Handling posture/length variations in the modular link. Because the forearm link is adjustable, changes in posture or link length slightly modify the robot’s internal weight distribution. In our platform the adjustable span is 10–18 cm, which shifts the link center of mass by approximately 1.5 cm—a small variation for the operating ranges considered. The proposed controller addresses this through two complementary parts: (i) a model-based feedforward compensator $\:C(x,x\dot{})+G\left(x\right)$ computed from the nominal dynamics to cancel the dominant gravity, Coriolis, and centrifugal effects; and (ii) a model-free impedance law that applies gentle restoring torques proportional to the tracking error. The second (impedance) layer inherently absorbs small modeling mismatches—such as minor CoM shifts due to posture/length changes—without requiring gain retuning. In practice, this two-layer scheme ensures stable and accurate tracking across the 10–18 cm adjustable range, maintaining robustness to minor mass-distribution variations.

## Results and discussion

### Simulation results

**Fig. 17:**
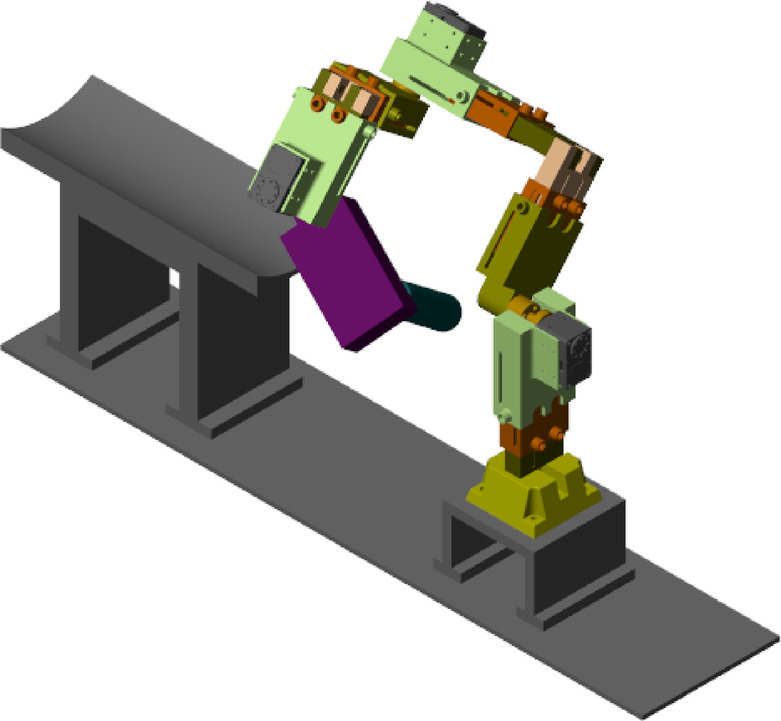
3D view of robot movement in Simscape MATLAB simulation environment.

Following the completion of component design in SolidWorks and the controller design, the robot’s performance was evaluated through comprehensive simulations in MATLAB/Simulink using Simscape Multibody. Simulink, with its user-friendly interface, provides an integrated environment for simulating both the physical model and control system, ensuring precision through the use of Simscape libraries. These tools enable the accurate modeling of dynamic systems and the effective implementation of control strategies. Figure 17 illustrates the simulated robot within the MATLAB environment.

To evaluate the dynamic performance of the proposed control scheme, simulation tests were performed on all three wrist degrees of freedom—pronation/supination (PS), abduction/adduction (AA), and flexion/extension (FE)—commanded simultaneously with sinusoidal reference trajectories:

$$\:{\theta\:}_{PS}=80^\circ\:\text{sin}\left(\frac{\pi\:}{2}t\right)$$

$$\:{\theta\:}_{AA}=20^\circ\:\text{sin}\left(\frac{\pi\:}{2}t\right)$$

$$\:{\theta\:}_{FE}=50^\circ\:\text{sin}\left(\frac{\pi\:}{2}t\right)$$

These motions emulate the controlled back-and-forth movements used in wrist rehabilitation exercises, enabling the assessment of coordinated tracking across all joints.

The control system combined the impedance law in task space with model-based dynamic compensation $\:C\left(x,\dot{x}\right)+G\left(x\right)$, mapped to joint torques through the Jacobian transpose. The impedance gains were selected as $\:{K}_{d}=2$ and $\:{C}_{d}=0.5$ after preliminary tuning, which provided a smooth yet responsive behavior without overshoot or instability. These values represent a compromise between sufficient stiffness for accurate trajectory tracking and low damping to preserve motion compliance.

**Fig. 18:**
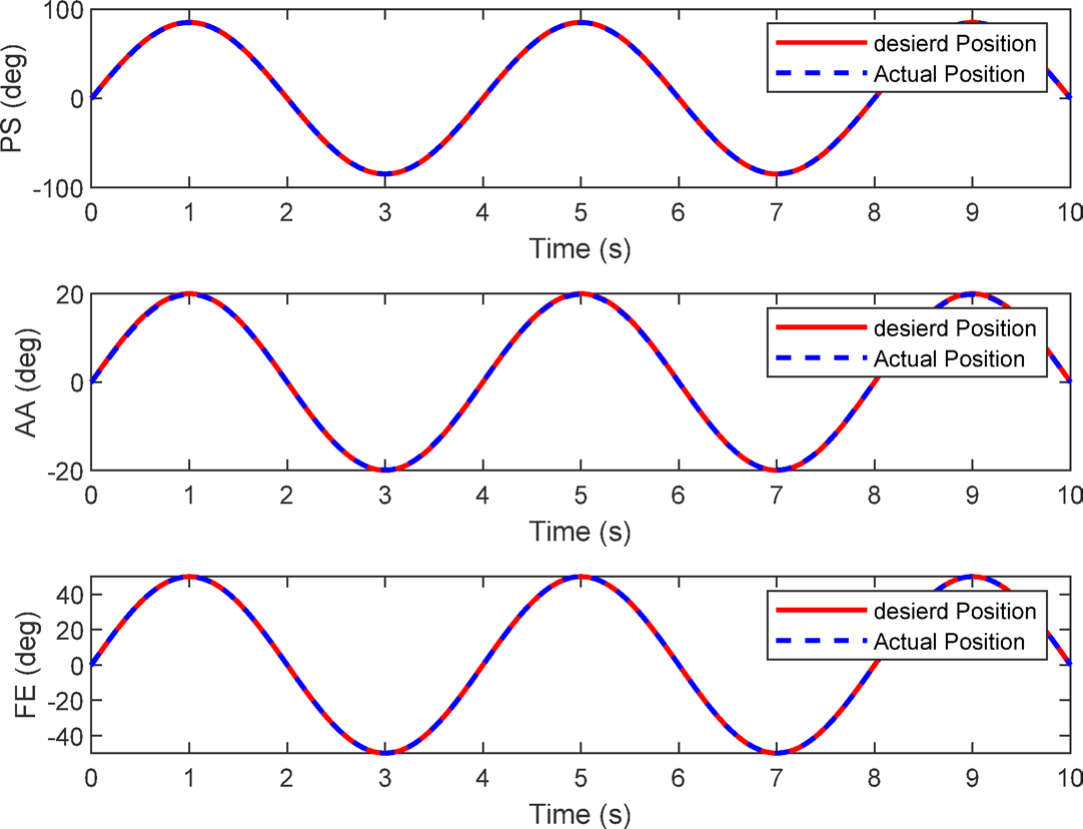
Robot response to impedance control and weight compensation for three movements.

**Fig. 19:**
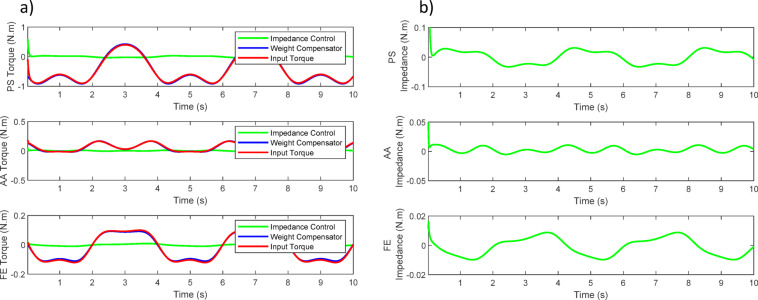
(**a**) Input torque of motors. (**b**) Input impedance torque.

Figure 18 illustrates the simulated joint positions during the combined motion. All three joints follow their sinusoidal references closely, showing synchronized and stable tracking over multiple cycles. Figure 19a presents the total commanded joint torques, representing the combined action of the impedance term and the dynamic compensation. The torque profiles remain periodic and smooth, within realistic actuator limits. Figure 19b isolates the impedance only torque component, which is notably smaller than the total torque. The results show that the weight-compensation term supplies the majority of the required torque, while the impedance law contributes a smaller corrective component that shapes the compliant interaction.

To further evaluate the effectiveness of the proposed weight-compensation strategy, two sets of comparative simulations were conducted: one using impedance control alone (Imp) and the other using impedance control with dynamic weight compensation (Imp + WC).

The same motion trajectories were applied in both cases to ensure fair comparison. The impedance gains were set to $\:{K}_{d}=6$ and $\:{C}_{d}=2$ for the Imp-only controller, and to $\:{K}_{d}=2$ and $\:{C}_{d}=0.5$ when the weight-compensator was active.

Three single-joint sinusoidal tests were performed to isolate the effect of compensation on each axis:

- Test 1: the PS joint follows an 80°·sin(πt/2) trajectory, while AA and FE are fixed.
- Test 2: the AA joint performs a 20°·sin(πt/2) motion, with PS and FE fixed.
- Test 3: the FE joint executes a 50°·sin(πt/2) trajectory, with PS and AA held constant.

**Fig. 20:**
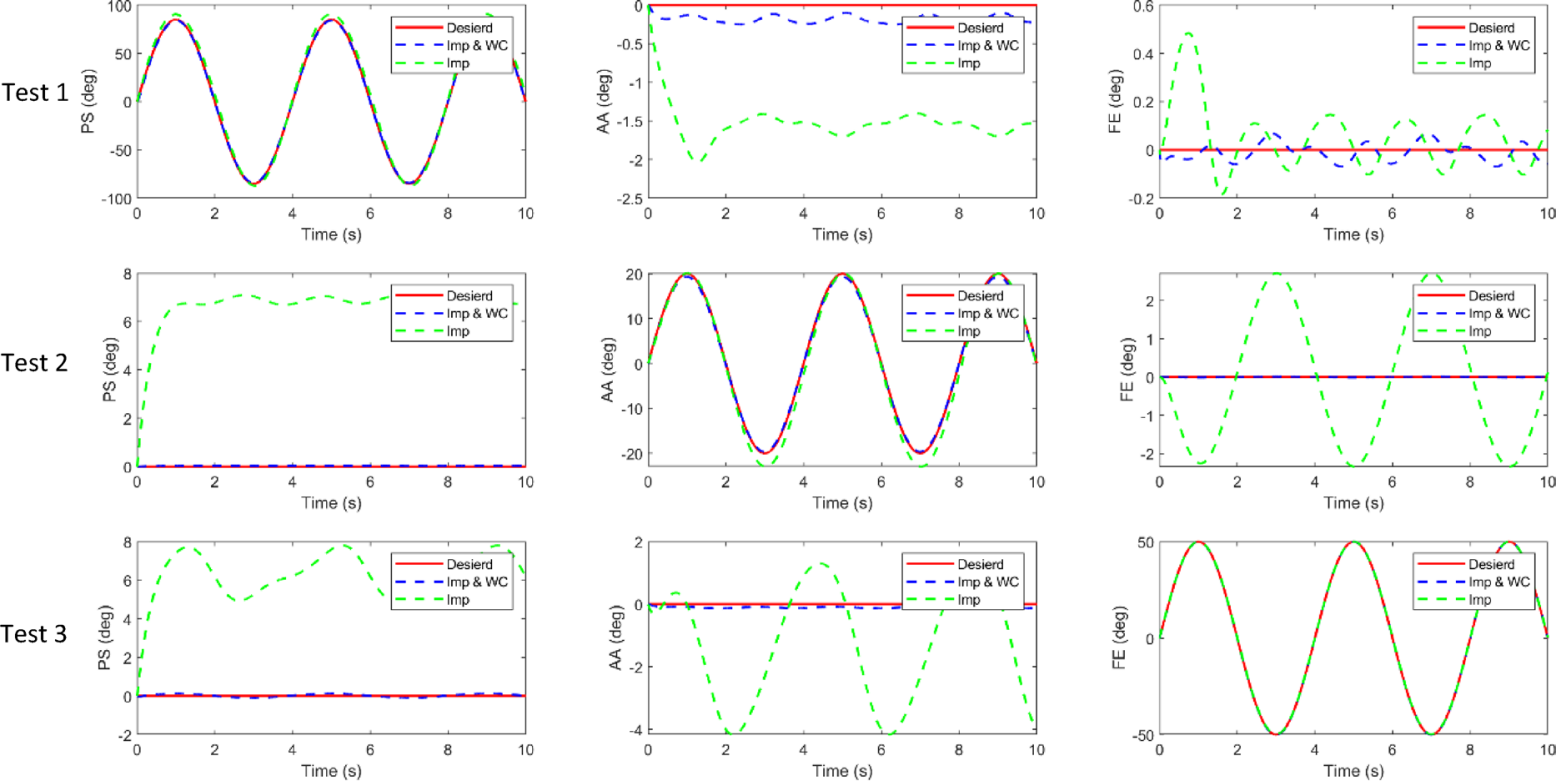
Joint position tracking under impedance control with and without weight compensation.

Figure 20 presents the joint position tracking results under both control schemes. As shown, without the weight compensator (green dashed lines), the joints exhibit steady-state offsets and oscillations, particularly in the PS and AA axes, which are more affected by gravity. When the compensator is activated (blue dashed lines), these deviations are almost eliminated, and the joints track their reference trajectories more accurately and smoothly.

**Fig. 21:**
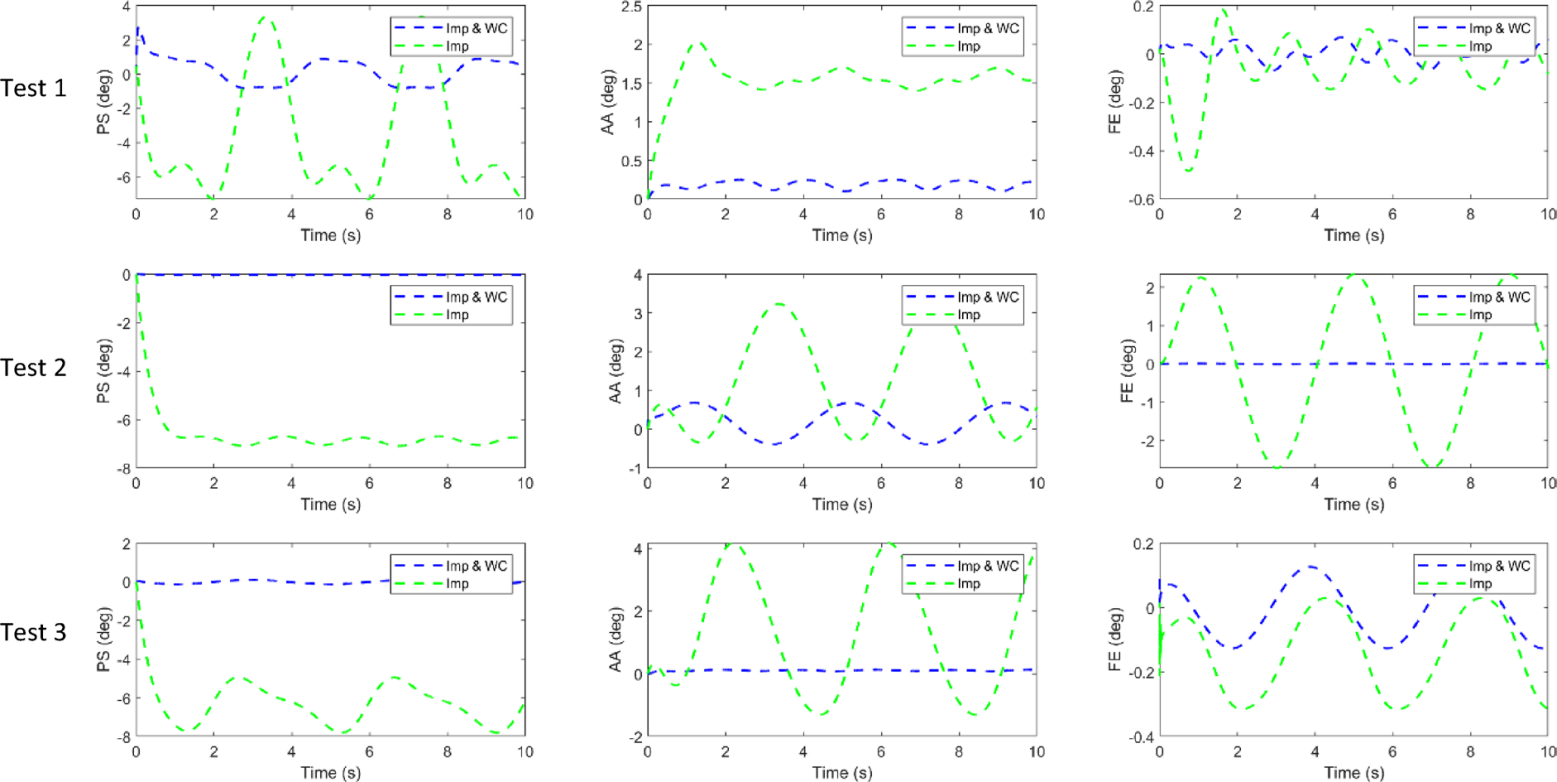
Tracking errors for the same three tests.

Figure 21 shows the corresponding tracking errors. The uncompensated controller displays higher amplitude error oscillations, while the compensated controller demonstrates substantially reduced RMS error and improved stability.

The quantitative results are summarized in Table 4, which lists the RMS tracking errors for all single-joint tests. The inclusion of the dynamic compensation term $\:C\left(x,\dot{x}\right)+G\left(x\right)$, markedly decreases the RMS error in every case—from approximately 5–6° to below 1° in the PS axis, and by comparable ratios for AA. As also evident, the improvement is less pronounced for the FE joint, which carries a smaller share of the manipulator’s gravitational load; consequently, its uncompensated error is inherently lower and the relative reduction with compensation is more modest than in PS and AA. These findings confirm that the compensator effectively cancels static and velocity-dependent torque components due to gravity, allowing the impedance controller to maintain precise and compliant joint motion.

**Table 4:** RMS error for simulation tests.

	Test	Position error for PS joint (deg)	Position error for AA joint (deg)	Position error for FE joint (deg)
Impedance control without weight compensator $\:{{K}}_{{d}}=6$ $\:{{C}}_{{d}}=2$	$\:PS\:=\:80\text{sin}\left(\frac{\pi\:}{2}t\right)$ $\:FE=AA=0$	$\:5$	$\:1.55$	$\:0.147$
	$\:AA\:=20\text{sin}\left(\frac{\pi\:}{2}\:t\right)$ $\:PS=FE=0$	$\:6.7$	$\:1.75$	$\:1.72$
	$\:FE\:=50\text{sin}\left(\frac{\pi\:}{2}t\right)$ $\:PS=AA=0$	$\:6.39$	$\:2.21$	$\:0.183$
Impedance control with weight compensator $\:{\varvec{K}}_{{d}}=2$ $\:{{C}}_{{d}}=0.5$	$\:PS\:=\:80\text{sin}\left(\frac{\pi\:}{2}t\right)$ $\:FE=AA=0$	$\:0.79$	$\:0.19$	$\:0.038$
	$\:AA\:=20\text{sin}\left(\frac{\pi\:}{2}t\right)$ $\:PS=FE=0$	$\:0.03$	$\:0.43$	$\:0.01$
	$\:FE\:=50\text{sin}\left(\frac{\pi\:}{2}t\right)$ $\:PS=AA=0$	$\:0.08$	$\:0.1$	$\:0.08$

### Experimental results

**Fig. 22:**
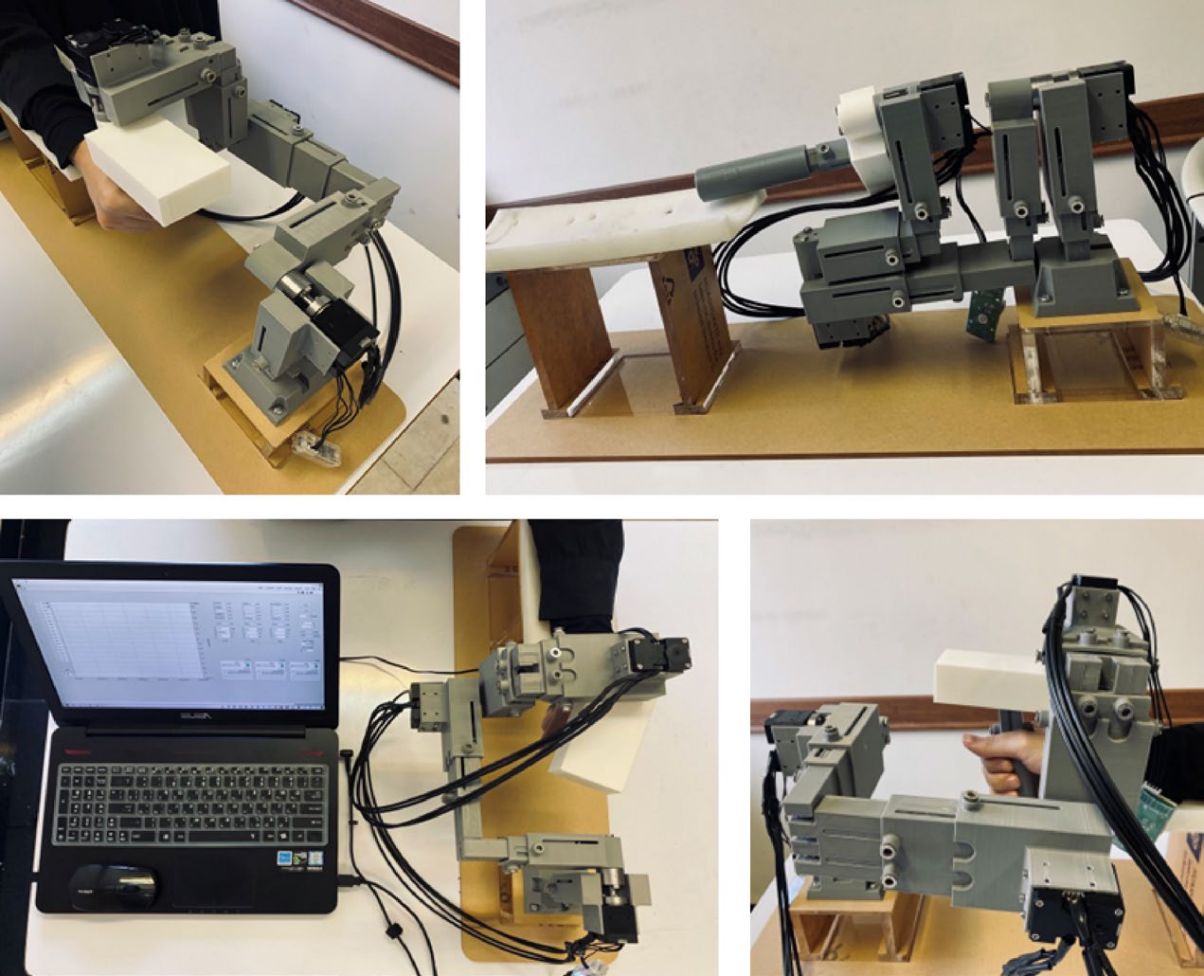
Final assembly of the wrist rehabilitation robot.

In this study, a prototype of the wrist rehabilitation robot was 3D printed using PLA material. Steel was chosen for the shafts due to its high strength and durability. The robot’s design was optimized for easy folding, reducing space requirements and facilitating transport. Figure 22 shows the built prototype of the robot. Dynamic analysis conducted using the (PS, AA, FE) configuration showed that for sinusoidal motion with a maximum angular velocity of $\:\frac{\pi\:}{2}$ rad/s^34^, the required torques for different movements were as follows: supination/pronation at least 1 Nm, abduction/adduction 0.5 Nm, and flexion/extension 0.2 Nm. Additionally, the motor must withstand a minimum radial force of 15 N·m. Therefore, the Dynamixel XM430-W350 motor was chosen as it meets these torque and force requirements.

To evaluate the practical performance of the modular wrist rehabilitation robot, a set of preliminary experiments was conducted with a healthy adult to verify the performance of both the device and its controller. No clinical or therapeutic data were collected at this stage. The participant was instructed to follow predefined sinusoidal trajectories displayed on a screen, which allowed consistent and repeatable testing of the robot’s tracking accuracy and stability.

In wrist rehabilitation, the aim is not to follow complex motion paths; rather, therapy prioritizes repetitive, controlled back-and-forth joint motions for the wrist. Accordingly, simple sinusoidal trajectories were used as test inputs because they faithfully reproduce these rehabilitation patterns.

Three single-DOF movements were tested: For the pronation/supination (PS) movement, the participant tracked a 50°sin(0.6π t) reference, requiring smooth rotation about the forearm axis. The abduction/adduction (AA) motion used 20°sin(0.6π t) to evaluate lateral wrist control. Finally, the flexion/extension (FE) movement followed 50°sin(0.6π t), assessing the robot’s assistance in upward–downward wrist bending.

The experimental results showed that the impedance controller successfully guided the user’s movements with safe and smooth motion. However, noticeable differences were observed between the simulation and experimental results due to the practical limitations of the hardware and control setup. In simulation, the impedance parameters were$\:{K}_{d}=\:2$ and $\:{C}_{d}\:=\:0.5$, whereas in the physical prototype these values were reduced to $\:{K}_{d}\:=\:0.5$ and $\:{C}_{d}\:=\:0$ to prevent instability. Thus, in the prototype, the controller was implemented with stiffness only (K-only).

This simplification was necessary due to several practical factors:

- *Prototype nature and PLA structure (limited rigidity and joint backlash).*
  Since this robot represents an early-stage prototype, its links were 3D-printed from PLA, resulting in limited structural rigidity and small backlash at the joints. These mechanical imperfections introduce micro-impacts during direction reversals and reduce the effective stiffness of the structure. When derivative or high-gain control terms are applied, these elastic effects are easily excited, leading to oscillations and a narrower stability margin.
- *Limited control-loop bandwidth (~ 100 Hz) and noisy velocity estimation.*
  The outer control loop operated at approximately 100 Hz, which is relatively low for implementing virtual damping or inertia in a stable manner. At this frequency, velocity must be estimated numerically from encoder position data, which is inherently noisy. Because damping and inertial terms depend directly on the accuracy of velocity and acceleration estimates, this noise causes fluctuating control torques and deteriorates stability.
- *Timing jitter under a non-real-time operating system (Windows).*
  The controller was executed under Windows, which lacks real-time scheduling guarantees. The resulting timing jitter behaves as a variable delay in the control loop. Derivative and virtual-inertial terms are particularly sensitive to such delays; even small, time-varying latencies can significantly reduce phase margin and lead to oscillatory behavior.
- *Unmodelled nonlinearities and actuator-level constraints.*
  Current and voltage limits, finite inner-loop bandwidth, gear compliance, and Coulomb/viscous friction are not fully captured by the nominal dynamic model. Introducing virtual damping or mass increases torque demands and sensitivity to velocity, which can push the actuators toward saturation and induce stick–slip behavior, further compromising stability.

As a result, although virtual damping and inertia were stable in simulation, they caused oscillations on the hardware. A simplified K-only impedance controller combined with model-based feedforward compensation $\:C\left(x,\dot{x}\right)+G\left(x\right)$provided the most stable and smooth performance.

**Fig. 23:**
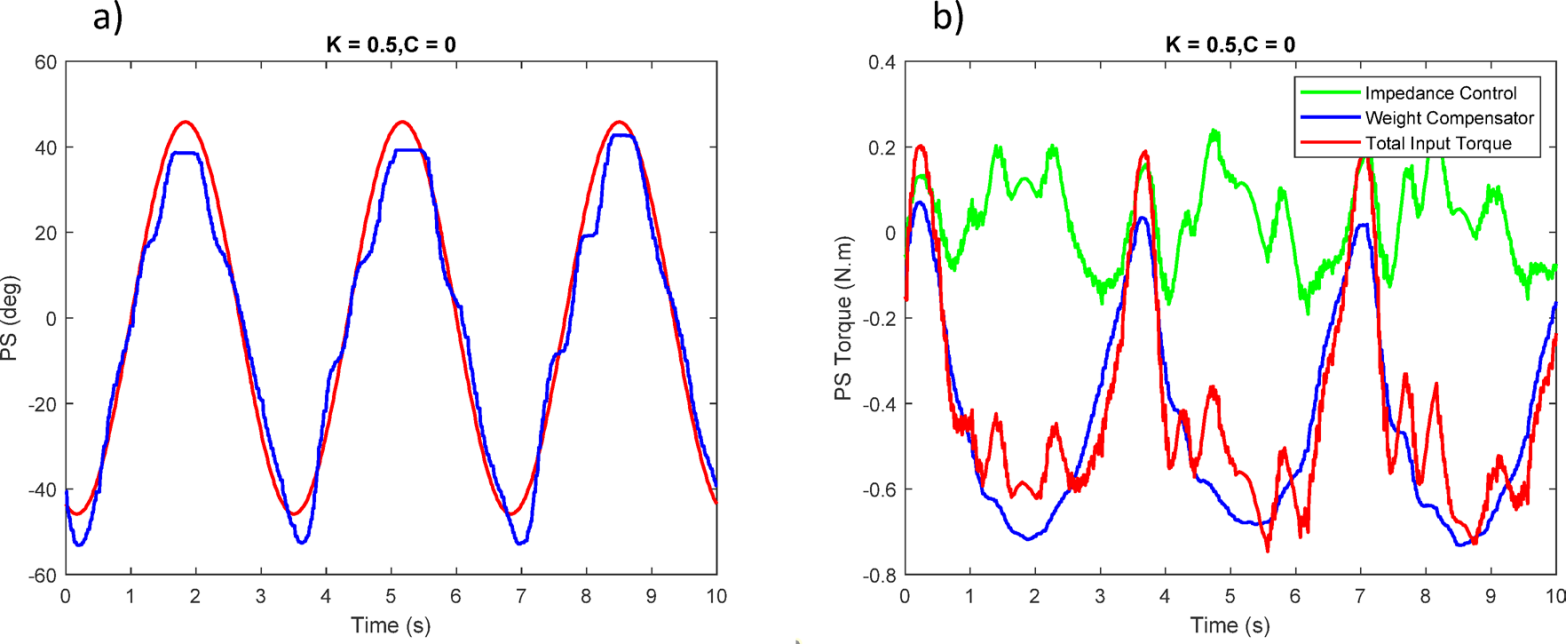
(**a**) Tracking of the first joint position with user assistance. (**b**) Torque applied to the first joint.

**Fig. 24:**
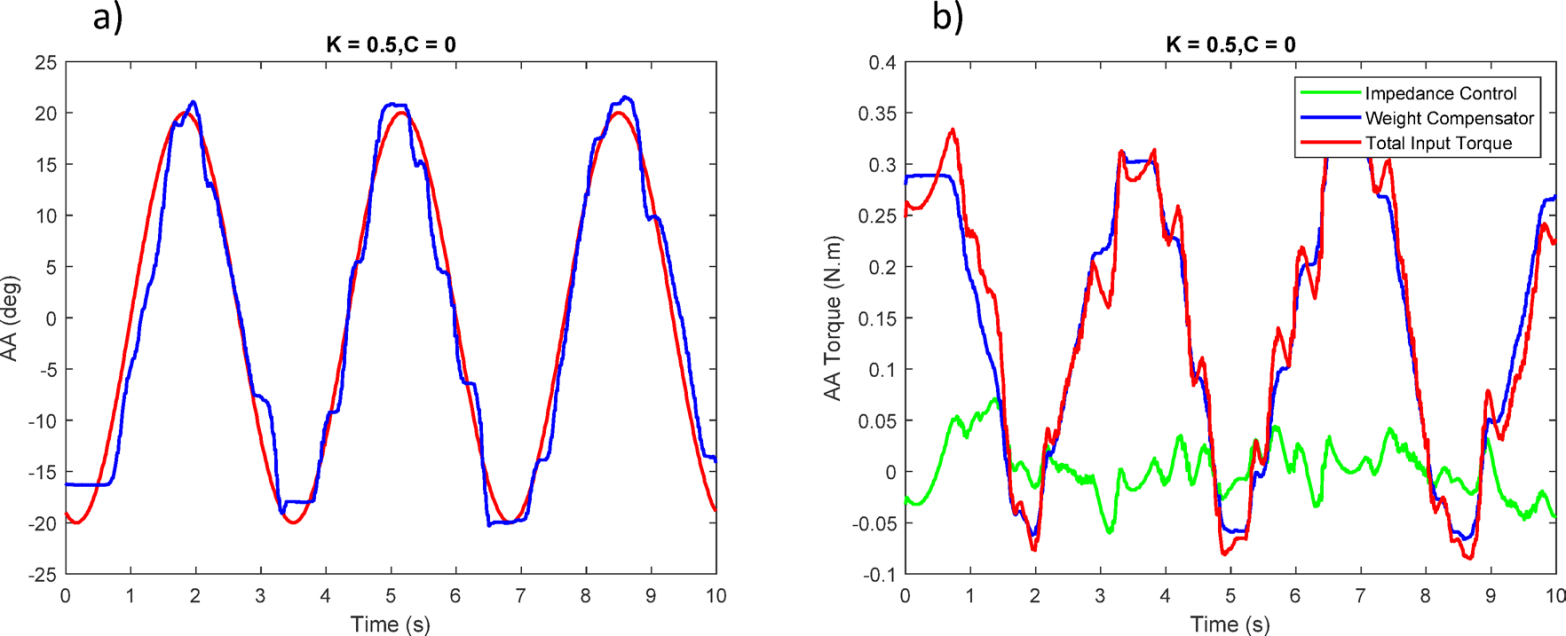
(**a**) Tracking of the second joint position with user assistance. (**b**) Torque applied to the second joint.

**Fig. 25:**
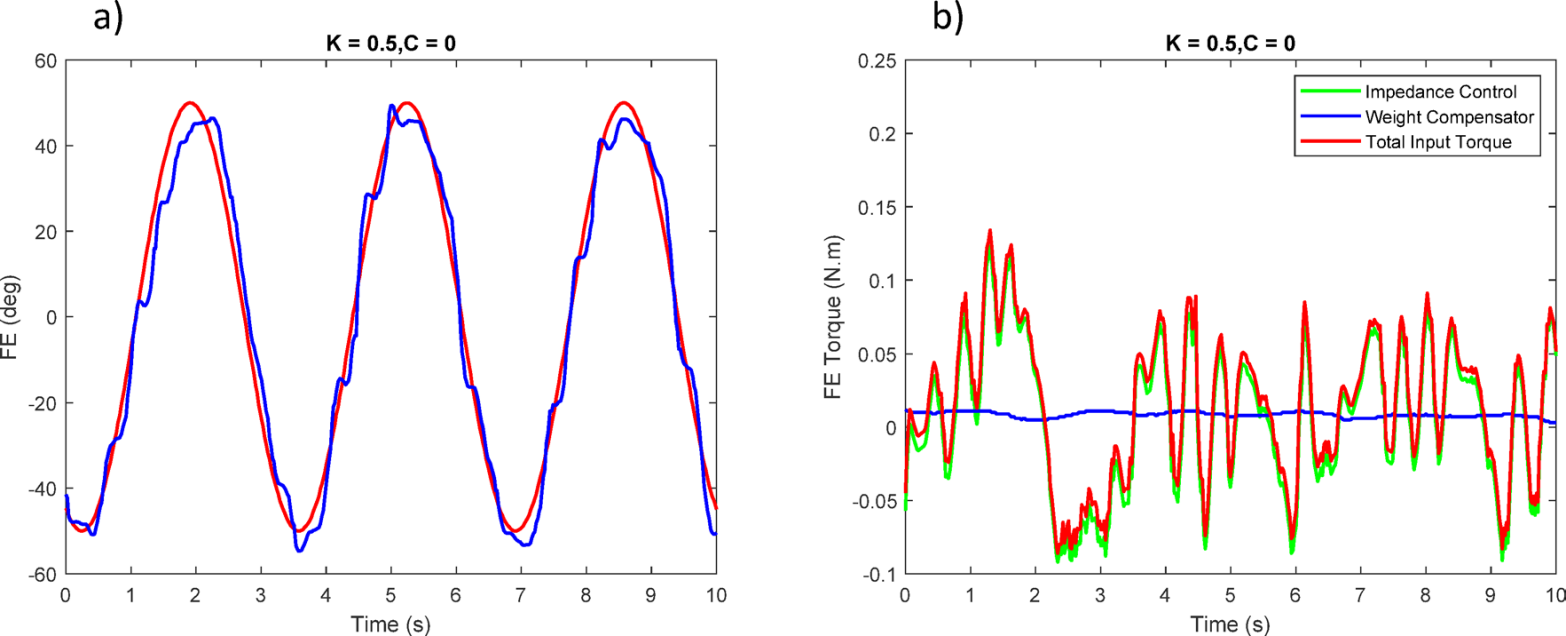
(**a**) Tracking of the third joint position with user assistance. (**b**) Torque applied to the third joint.

Figures 23, 24, 25 report the tracking performance and the corresponding torque responses for the three wrist movements. Although the motors are not equipped with torque sensors, the joint torques shown were estimated from motor current feedback using the known torque constant ($\:\tau\:=Ki)$.

Overall, the experimental results confirm that the proposed control strategy ensures stable, safe, and adaptive wrist motion. Although no dedicated force/torque sensor was installed at the human–robot interface, interaction was inferred from the servo motor torque feedback within the impedance-control framework. When the participant accurately follows the reference sinusoidal trajectory, the controller records minimal corrective torque (i.e., no meaningful resistive action from the robot); small deviations from the desired path elicit gentle restoring torques proportional to the error, guiding the wrist back toward the intended motion without discomfort. This proxy effectively captures the compliant and adaptive behavior of the controller during rehabilitation exercises.

While the experiments used simple sinusoidal movements, this design choice aligns with the actual goals of wrist rehabilitation, where repetitive and controlled joint motions are essential for restoring flexibility and muscle coordination. Future work will involve real-time torque sensing and user-based comfort evaluations to further validate the clinical applicability of the proposed system.

## Conclusion

The modular wrist rehabilitation robot developed in this study integrates the key tenets of modular architecture. It comprises detachable modules that can be readily replaced or swapped, providing flexibility for diverse rehabilitation tasks. Standardized connectors enable seamless assembly, and module replacement can be performed quickly without specialized expertise. This modularity also facilitates future upgrades and the addition of new modules to meet evolving clinical needs. The mechanism supports multiple degrees of freedom, enhancing adaptability to varied patient requirements.

On the control side, a task-space impedance law was combined with model-based weight/dynamic compensation. Simulation studies validated the approach: impedance alone did not yield optimal performance, whereas including compensation markedly reduced tracking error (RMS decreased from 6° to 0.8°) and allowed lower gains without sacrificing accuracy. Overall, the experimental results confirm that the proposed control strategy ensures stable, safe, and adaptive wrist motion. Although no direct force/torque sensor was employed, human–robot interaction is inherently reflected in the impedance torque response: when the participant follows the reference sinusoid (representative of rehabilitation practice), the controller applies virtually no resistive torque; small deviations elicit gentle restoring torques proportional to error, guiding the wrist back toward the intended motion without discomfort or excessive load. In sum, the modular hardware combined with the advanced control scheme offers a highly adaptable, efficient, and patient-centered solution for wrist rehabilitation.

Several limitations were identified. First, the control loop operated at a fixed ~ 100 Hz without the ability to increase the rate, constraining motion accuracy. Deploying a real-time operating system (RTOS) with deterministic scheduling (fixed, pre-defined intervals) is expected to improve control precision. Second, the robot becomes unbalanced when motors are de-energized, reducing passive stability; symmetry in link design and/or integrating joint springs and dampers^39^ could mitigate this during motor shutdown. Third, this study evaluated only the robot’s dynamics and did not model hand impedance; future work should estimate hand dynamics and simulate coupled human–robot motion for a more comprehensive assessment. Finally, no clinical trials were conducted here; subsequent studies will include multi-participant testing and clinical evaluations to quantify interaction forces and confirm safety and efficacy for long-term use.

## Supplementary Information

Below is the link to the electronic supplementary material.

Supplementary Material 1

## Data Availability

The experimental data obtained in this study have been provided as a supplementary file entitled “Experimental Tests.” Furthermore, additional supporting data not included in the supplementary file are available from the corresponding author upon reasonable request.
